# The Association of Amyloid‐β and Tau With Synaptic Dysfunction in Alzheimer's Disease

**DOI:** 10.1002/acn3.70558

**Published:** 2026-10-30

**Authors:** Fausto Roveta, Augusto J. Mendes, Valentina Garibotto, Cecilia Boccalini, Aurelien Lathuiliere, Innocenzo Rainero, Elisa Rubino, Henrik Zetterberg, Nicholas J. Ashton, Federica Ribaldi, Giovanni B. Frisoni, for the Alzheimer's Disease Neuroimaging Initiative

**Affiliations:** ^1^ Department of Neuroscience “Rita Levi‐Montalcini” University of Turin Turin Italy; ^2^ Geneva Memory Center, Department of Rehabilitation and Geriatrics Geneva University Hospitals Geneva Switzerland; ^3^ Laboratory of Neuroimaging of Aging (LANVIE) University of Geneva Geneva Switzerland; ^4^ Division of Nuclear Medicine and Molecular Imaging, Diagnostic Department Geneva University Hospitals Geneva Switzerland; ^5^ Laboratory of Neuroimaging and Innovative Molecular Tracers (NIMTlab), Geneva University Neurocenter and Faculty of Medicine University of Geneva Geneva Switzerland; ^6^ CIBM Center for Biomedical Imaging Geneva Switzerland; ^7^ Laboratory for Translational Research in Neurodegeneration University of Geneva Geneva Switzerland; ^8^ Department of Psychiatry and Neurochemistry, Institute of Neuroscience & Physiology The Sahlgrenska Academy at the University of Gothenburg Mölndal Sweden; ^9^ Clinical Neurochemistry Laboratory, Sahlgrenska University Hospital Mölndal Sweden; ^10^ Department of Neurodegenerative Disease UCL Institute of Neurology London UK; ^11^ Department of Pathology and Laboratory Medicine University of Wisconsin School of Medicine and Public Health Madison Wisconsin USA; ^12^ Wisconsin Alzheimer's Disease Research Center, University of Wisconsin School of Medicine and Public Health University of Wisconsin‐Madison Madison Wisconsin USA; ^13^ UK Dementia Research Institute at UCL London UK; ^14^ Banner Alzheimer's Institute and University of Arizona Phoenix Arizona USA; ^15^ Banner Sun Health Research Institute Sun City Arizona USA

## Abstract

**Objective:**

To clarify the relative contributions of amyloid and tau to synaptic dysfunction in Alzheimer's disease (AD).

**Methods:**

We included 109 cognitively unimpaired and impaired individuals from the Geneva Memory Center with available CSF synaptic biomarkers (neurogranin, SNAP‐25, GAP‐43). Participants were stratified according to amyloid (A) and tau (T) status, and group comparisons and correlations between synaptic and AD biomarkers were assessed. Multiple linear regression models evaluated the independent associations of amyloid and tau with synaptic markers: Model I (*n* = 109) included CSF Aβ42 and p‐tau181, while Model II (*n* = 69) included amyloid PET centiloid and CSF p‐tau181. Mediation analyses were performed to further investigate these relationships. Findings were replicated in the ADNI cohort (*n* = 787).

**Results:**

Synaptic biomarker levels were significantly higher in A+/T+ individuals compared with A−/T− and A+/T− groups (*p* < 0.001). CSF p‐tau181 strongly correlated with synaptic markers. In Model I, GAP43 was positively associated with both CSF Aβ42 (*β* = 0.31, 95% CI 0.23–0.40) and p‐tau181 (*β* = 0.94, 95% CI 0.84–1.04; *p* < 0.001). In Model II, lower amyloid PET centiloid (*β* = −0.31, 95% CI −0.46 to −0.16) and higher p‐tau181 (*β* = 1.02, 95% CI 0.86–1.19) predicted increased GAP43 (*p* < 0.001), with the same pattern of associations observed for neurogranin and SNAP‐25. The association between amyloid burden and synaptic biomarkers was largely indirect, statistically mediated by CSF p‐tau181. Results were replicated in ADNI.

**Interpretation:**

Tau pathology showed strong associations with synaptic biomarkers, with a potential mediating role in the association between amyloid pathology and synaptic dysfunction. These findings support a central role of tau‐related processes in synaptic dysfunction in AD.

## 1 Introduction

Alzheimer's disease (AD) is neuropathologically defined by the accumulation of extracellular amyloid‐β (Aβ) plaques and intracellular neurofibrillary tangles composed of hyperphosphorylated tau. These pathological changes begin years or even decades before the onset of clinical symptoms [1]. Despite their defining role, the burden of amyloid plaques and tau tangles shows only partial correlations with cognitive impairment [2]. In contrast, disruption of synaptic integrity represents a pivotal downstream event, and in vivo imaging and neuropathological studies consistently demonstrate that synapse loss is the strongest correlate of cognitive impairment in AD [3, 4].

Recent advances in cerebrospinal fluid (CSF) biomarker research have enabled the in vivo assessment of synaptic integrity in AD [5]. Many synaptic biomarkers have been proposed, each with potential distinct characteristics, such as varying susceptibility to AD pathology [6]. Because presynaptic and postsynaptic compartments show distinct vulnerability in AD, assessing proteins from both compartments may capture complementary aspects of synaptic damage. Neurogranin, a postsynaptic protein involved in synaptic plasticity, has consistently been shown to be elevated in AD and to reflect dendritic spine pathology [7]. Synaptosomal‐associated protein 25 kDa (SNAP‐25), a presynaptic protein essential for vesicle fusion and neurotransmitter release, has emerged as another sensitive biomarker of presynaptic dysfunction [8]. Growth‐associated protein 43 (GAP‐43), highly expressed in neuronal growth and synaptogenesis, is enriched in regions most vulnerable to AD pathology and detectable at increased concentrations in CSF of affected individuals [9].

Accumulating experimental evidence indicates that synaptic damage is linked to both Aβ and tau pathology [10, 11]. Aβ is thought to trigger aberrant neuronal and synaptic activity at early disease stages, thereby creating a permissive environment for tau hyperphosphorylation, propagation, and aggregation, which in turn further amplifies synaptic injury [12, 13, 14, 15]. Consistently, longitudinal studies have shown that increased CSF levels of neurogranin, SNAP‐25, and GAP‐43 are associated with accelerated brain atrophy, hypometabolism, and faster cognitive decline, particularly in amyloid‐positive individuals [16, 17, 18]. More recent observations further suggest that synaptic functional alterations may facilitate the trans‐neuronal spread of tau along functionally connected networks [19]. Within this framework, Aβ‐induced synaptic hyperactivity could promote the synaptic release and propagation of hyperphosphorylated tau, thereby linking early amyloid pathology to downstream tau spreading through synaptic mechanisms [20].

Overall, these observations support a close interrelationship between amyloid, tau and synaptic damage.

While some studies have reported a predominant association between synaptic biomarkers and Aβ burden, suggesting that synaptic damage may already be detectable in amyloid‐positive individuals in the absence of significant tau pathology [21], others have described a stronger relationship with tauopathy [11].

Although Aβ and tau together represent the neuropathological signature of Alzheimer's disease, they reflect partially distinct pathophysiological mechanisms. This is supported by their different spatial distributions across the brain and by the divergent cognitive trajectories observed in individuals with significant amyloid‐β deposition but not tauopathy compared with those in whom both proteinopathies coexist [22, 23]. Yet, the in vivo independent effect of Aβ and tau in determining synaptic damage, which is thought to be one of the earliest and clinically relevant events of neuronal injury in AD, remains unresolved. Against this background, the present study aims to investigate the relationships between Aβ, tau and synaptic damage biomarkers in a cohort of individuals attending our tertiary Memory Clinic and to validate our findings with an independent cohort.

## 2 Subjects/Materials and Methods

### 2.1 Population

The study included 109 subjects retrospectively selected from the Geneva Memory Center (GMC) of the Geneva University Hospitals from 2014 to 2022. The GMC cohort includes individuals ranging from cognitively unimpaired (CU) subjects, either healthy volunteers or individuals with subjective cognitive decline (SCD), to patients with mild cognitive impairment (MCI) and dementia determined according to the Clinical Dementia Rating (CDR) score (CDR = 0, cognitively unimpaired; CDR = 0.5, MCI; CDR ≥ 1, dementia), as previously described [24]. The inclusion criteria for this study were: availability of (i) amyloid PET or CSF Aβ42 data, and (ii) tau PET or CSF p‐tau181 levels, and (iii) CSF synaptic biomarkers (neurogranin, SNAP‐25, GAP‐43) data. Amyloid PET was available for 69 individuals, and tau PET for 48 individuals. All participants had CSF biomarkers available; the availability of PET data across A/T groups is reported in Table 1.

**TABLE 1:** Demographics, cognitive stages, and biomarkers status of subjects by study groups (GMC).

Variable	A−/T− (*N* = 52)	A+/T− (*N* = 30)	A+/T+ (*N* = 27)	*p*
Demographics				
Age (years) (mean ± SD)	66 ± 8^b^	71 ± 8	72 ± 6^a^	0.018
Gender, female (%)	46	37	67	0.068
Education (years) (mean ± SD)	14 ± 4	14 ± 4	15 ± 3	0.886
Cognitive stages and global scores				
CU/MCI/Dementia (%)	72/23/4^a^	32/61/7^b^	15/63/22^b^	< 0.001
Mini‐Mental State Examination (mean ± SD)	28.2 ± 2.2^a^	27.0 ± 2.3^b^	25.0 ± 4.2^b^	< 0.001
PET imaging				
Amyloid PET Centiloid (mean ± SD, *N*)	−0.5 ± 6.1^b^ (*n* = 30)	62.4 ± 24.4^a^ (*n* = 20)	78.8 ± 26.9^a^ (*n* = 19)	< 0.001
Tau PET global meta‐ROI (mean ± SD; *N*)	1.20 ± 0.07^b^ (*n* = 19)	1.27 ± 0.24^b^ (*n* = 15)	1.66 ± 0.41^a^ (*n* = 14)	< 0.001
CSF biomarkers				
Aβ42 (mean ± SD)	1103 ± 327^a^	599 ± 208^b^	527 ± 161^b^	< 0.001
p‐Tau181 (mean ± SD)	41 ± 16^b^	53 ± 25^b^	99 ± 37^a^	< 0.001
t‐Tau (mean ± SD)	292 ± 130^b^	342 ± 185^b^	706 ± 321^a^	< 0.001
GAP‐43 (mean ± SD)	3106 ± 1276^b^	2874 ± 1204^b^	4750 ± 1751^a^	< 0.001
Neurogranin (mean ± SD)	153 ± 59^b^	144 ± 59^b^	248 ± 98^a^	< 0.001
SNAP‐25 (mean ± SD)	160 ± 51^b^	151 ± 55^b^	237 ± 81^a^	< 0.001

*Note:* Numerical data are presented as the mean (standard deviation), while categorical data are expressed as the number (percentage of the total). For numerical variables, *p*‐values were calculated using the Kruskal–Wallis test, with post hoc analyses conducted using the Mann–Whitney *U* test. For categorical variables, *p*‐values were derived from contingency table analyses using the *χ* ^2^ test. Post hoc comparisons are reported as a > b > c.

SCD has been defined as self‐reported cognitive impairment with normal neuropsychological assessment results. MCI has been defined by objective cognitive deficits, and maintenance of daily functioning. Dementia was diagnosed in individuals who met the criteria for cognitive impairment but also showed impairment in activities of daily living. Neuropsychological tests included the Mini‐Mental State Examination (MMSE) as a measure of global cognition. More details on procedures of clinical and cognitive assessments are provided elsewhere [24].

As an independent validation cohort, we used data from the Alzheimer's disease neuroimaging initiative (ADNI) dataset [25].

Data used in the preparation of this article were obtained from the ADNI database (adni.loni.usc.edu). The ADNI was launched in 2003 as a public‐private partnership, led by Principal Investigator Michael W. Weiner, MD. The primary goal of ADNI has been to test whether serial magnetic resonance imaging (MRI), PET, other biological markers, and clinical and neuropsychological assessment can be combined to measure the progression of MCI and early AD.

For this study, we selected all participants with available CSF GAP‐43; neurogranin and SNAP‐25 were acquired in ADNI to different timelines and were not concurrently available within this cohort; therefore, validation analyses were restricted to GAP‐43. All CSF Aβ42, CSF p‐tau181 and amyloid PET available data were included to replicate analyses from the GMC cohort. Tau PET was not used in the ADNI validation analyses because it was available only in a subset of participants and had been acquired more than 1 year after the lumbar puncture used for GAP‐43 measurement; to avoid temporal mismatch, and consistent with the GMC cohort, in which only PET examinations performed within 1 year of lumbar puncture were considered, T status in ADNI was defined using CSF p‐tau181.

### 2.2 Imaging and Biological Samples Protocols

#### 2.2.1 PET

For participants from the GMC cohort, acquisitions were performed on Siemens Biograph and Biograph Vision scanners (Siemens, Washington, DC) and reconstructed using 3D OSEM iterative reconstruction, with corrections for randoms, dead time, normalization, scatter, attenuation, and sensitivity. For amyloid PET ^18^F‐florbetapir or ^18^F‐flutemetamol tracers were used. For tau PET, ^18^F‐flortaucipir, synthesized at the Centre for Radiopharmaceutical Sciences in Villigen, Switzerland, under license from the intellectual property owner (Avid subsidiary of Lilly), was used. Images were acquired following previously described procedures [26]. Amyloid PET images were converted to standardized uptake value ratios (SUVRs) using the whole cerebellum as a reference region. The average SUVR was converted to Centiloid units in order that data from different radiotracers could be compared [27]. The median interval (IQR) between amyloid PET scan and lumbar puncture for participants included in the study was 1 (7) months. Tau PET images were converted to SUVR values using the cerebellar crus as a reference region [28]. We considered the global SUVR calculated from the entorhinal cortex, lateral occipital cortex, inferior temporal cortex, and amygdala, hereafter referred to as the global tau PET meta‐ROI SUVR, previously validated [29]. The median interval (IQR) between tau PET scan and lumbar puncture for participants included in the study was 2 (8) months.

#### 2.2.2 Biological Samples

CSF and blood samples were collected following GMC standardized procedures previously described. Briefly, CSF was obtained by lumbar puncture with an atraumatic needle after an overnight fast, gathered in polypropylene tubes, centrifuged within an hour at 2000 g for 10 min at room temperature, then aliquoted into polypropylene vials and frozen at −80°C. Levels of Aβ42 and p‐tau181 in CSF were measured using the enzyme‐linked immunosorbent assay (ELISA) with INNOTEST at a clinical chemistry laboratory of Geneva University Hospitals.

CSF neurogranin and GAP‐43 concentrations were measured at the University of Gothenburg with in‐house enzyme‐linked immunosorbent assays as previously described in detail [21]. CSF SNAP‐25 concentration was measured using an immunoprecipitation mass spectrometry method, as previously described [21].

DNA was extracted from blood of the plasma EDTA tubes using the automated QIAsymphony DSP DNA Kit #937255 (Qiagen, Venlo, Netherlands). *APOE* genotyping was performed using TaqMan genotyping MasterMix assay #4371355 (Thermo Fisher, Waltham, MA). *APOE* genotype status was available for 96 individuals.

### 2.3 Classification of Subjects in Biological Groups

Patients were classified according to their biomarker status, based either on PET or CSF Alzheimer's biomarker.

Participants from the GMC cohort without cognitive impairment and patients with mild cognitive impairment or dementia were stratified according to amyloid (A) and tau (T) into A−/T−, A+/T− or A+/T+, prioritizing PET‐based biomarkers over CSF if multiple Alzheimer's biomarker modalities were available. Amyloid positivity and tau Braak stages were visually assessed by an expert nuclear medicine physician (VG), acting as a single reader, following the standard operating procedures approved by the European Medicines Agency [30], and published recommendations [31]. For tau PET imaging, visual classification was adapted such that scans were considered positive at Braak stages IV–VI, as previously proposed [32]. When PET data were unavailable, A/T status was determined using previously validated, cohort‐specific CSF thresholds, classifying participants as A−/T−, A+/T−, or A+/T+ [33]. No A−/T+ individuals were identified in the GMC cohort.

Participants in ADNI were stratified into A−/T−, A+/T−, A+/T+, or A−/T+ using amyloid PET for the A criterion and p‐tau181 in cerebrospinal fluid for the T criterion. Processing methods of amyloid PET (^18^F‐florbetapir) can be found on LONI in the UC Berkeley AV45 Methods document. To determine the amyloid positivity in ADNI participants, we considered the cutoff of 1.11 using the whole cerebellum as a reference region, as recommended by the authors of the analysis. This cutoff corresponds to the upper bound of the 95% CI above the mean of younger healthy controls [34]. In the ADNI cohort, T categories were identified through previously published thresholds using CSF p‐tau181 [35].

### 2.4 Statistical Analysis

Statistical analyses were performed using SPSS version 29.0.1.0 and R version 4.5.1 (2025).

Baseline differences in demographic characteristics, clinical features, cognitive performance, and biomarker levels across A/T groups were assessed using the Kruskal–Wallis test for continuous variables and the *χ* ^2^ test for categorical variables. When appropriate, post hoc pairwise comparisons were performed using the Mann–Whitney *U* test with Bonferroni correction. Correlations between synaptic biomarkers and CSF Aβ42, CSF p‐tau181, amyloid PET Centiloid values (GMC), and global tau PET SUVR were evaluated using Spearman's rank coefficients.

To investigate the independent associations of amyloid‐β and tau pathology with synaptic biomarkers, separate multiple linear regression models were fitted with a different synaptic biomarker as the dependent variable. Two primary regression models were specified. In the first model, CSF Aβ42 and CSF p‐tau181 were entered simultaneously as predictors of CSF GAP‐43. In the second model, amyloid PET Centiloid values and CSF p‐tau181 were included as predictors of CSF GAP‐43. For each model, analyses were restricted to participants with complete data for all included variables (CSF model: *n* = 109; amyloid PET model: *n* = 69). Regression models were estimated using ordinary least squares (OLS). Model performance was quantified using the adjusted *R* ^2^ and the Akaike Information Criterion (AIC). To account for potential violations of homoscedasticity, statistical inference was based on heteroskedasticity‐consistent standard errors using the HC3 estimator. Multicollinearity among predictors was assessed using variance inflation factors (VIF). Model assumptions were evaluated using residual diagnostics, including assessment of residual normality with the Shapiro–Wilk test and heteroskedasticity with the studentized Breusch–Pagan test. These diagnostics informed the choice of robust inference but did not affect the interpretation of model coefficients. To visualize the independent contributions of amyloid‐related biomarkers and CSF p‐tau181 to CSF GAP‐43 levels, partial residual plots were generated by regressing both the dependent variable and each predictor of interest on the complementary covariate and plotting the resulting residuals. Primary models were restricted to the amyloid and tau predictors of interest. As sensitivity analyses, multiple linear regression models were repeated including age, education, and APOE ε4 status as covariates. In the GMC cohort, additional sensitivity analyses were performed (i) using CSF t‐tau instead of p‐tau181, both as predictor (adjusting for age, sex and *APOE* ε4 status) and as mediator, and (ii) further adjusting for age, sex and global cognition (MMSE) as an index of disease severity. In the ADNI cohort, primary models were also repeated in amyloid‐positive participants only to address potential disease‐status confounding.

To examine whether the positive associations between amyloid PET values and synaptic biomarker levels were mediated by p‐Tau181 levels, we conducted mediation analysis based on the expression ‘X → Y, mediated by M’ using PROCESS macro for SPSS (model Type 4). Indirect effects were tested using nonparametric bootstrap confidence intervals based on 5000 resamples with a 95% confidence level. Heteroscedasticity‐consistent standard errors (HC3) were used.

All analyses were replicated in the ADNI cohort with GAP‐43 as synaptic biomarker to validate findings. Statistical significance was defined as *p* < 0.05. The primary hypotheses concerned the independent associations of amyloid and tau biomarkers with GAP‐43 (Models I and II); analyses of neurogranin and SNAP‐25, tau PET subgroup analyses and sensitivity analyses were considered secondary. No formal correction for multiple testing was applied; however, all principal associations with CSF p‐tau181 (*p* < 0.001) would remain significant after Bonferroni correction for the number of regression models tested, whereas significant results with *p* values between 0.01 and 0.05 should be regarded as exploratory.

## 3 Results

A total of 109 participants from the GMC cohort (mean age 68.9 ± 8.2 years, 49% female) were included in this study. Characteristics of the cohort stratified by amyloid and tau status are shown in Table 1. Table S1 reports demographics, clinical characteristics, and biomarker profiles of included participants from the ADNI cohort (*n* = 787).

### 3.1 Synaptic Biomarkers Are Increased in Individuals With High Tau

#### 3.1.1 GMC Cohort

No significant correlations were found with CSF Aβ42 and synaptic biomarkers. Significant correlations were found between Aβ PET Centiloid and neurogranin (*ρ* = 0.25, *p* = 0.013), SNAP‐25 (*ρ* = 0.28, *p* = 0.020) or GAP‐43 (*ρ* = 0.30, *p* = 0.042). Neurogranin, SNAP‐25 and GAP‐43 were strongly correlated (*ρ* = 0.83, 0.80 and 0.78, respectively) with p‐tau181 (*n* = 109, *p* < 0.001). Tau PET meta‐ROI SUVR moderately correlated with neurogranin (*ρ* = 0.36, *p* = 0.013) and GAP‐43 (*ρ* = 0.40, *p* = 0.034), but not with SNAP‐25 (*ρ* = 0.26, *p* = 0.10). Scatterplots of GAP‐43 and Aβ PET Centiloid or p‐tau181 are shown in Figure S1.

The three synaptic biomarkers differed significantly across the A−/T− (*n* = 52), A+/T− (*n* = 30), and A+/T+ (*n* = 27) groups. Specifically, neurogranin values were comparable between A−/T− (153 ± 59 pg/mL) and A+/T− (144 ± 59 pg/mL), while they were increased in A+/T+ (248 ± 98 pg/mL; *H(2)* = 24.652, *p* < 0.001). A similar pattern was observed for SNAP‐25, with lower levels in A−/T− (160 ± 51 pg/mL) and A+/T− (151 ± 55 pg/mL), and higher concentrations in A+/T+ (237 ± 81 pg/mL; *H(2)* = 21.966, *p* < 0.001). For GAP‐43, levels were lowest in the A−/T− group (3106 ± 1276 pg/mL) and in the A+/T− group (2874 ± 1204 pg/mL) compared to the A+/T+ group (4750 ± 1751 pg/mL; *H(2)* = 20.590, *p* < 0.001).

An overview of these findings is presented in Figure 1.

**FIGURE 1:**
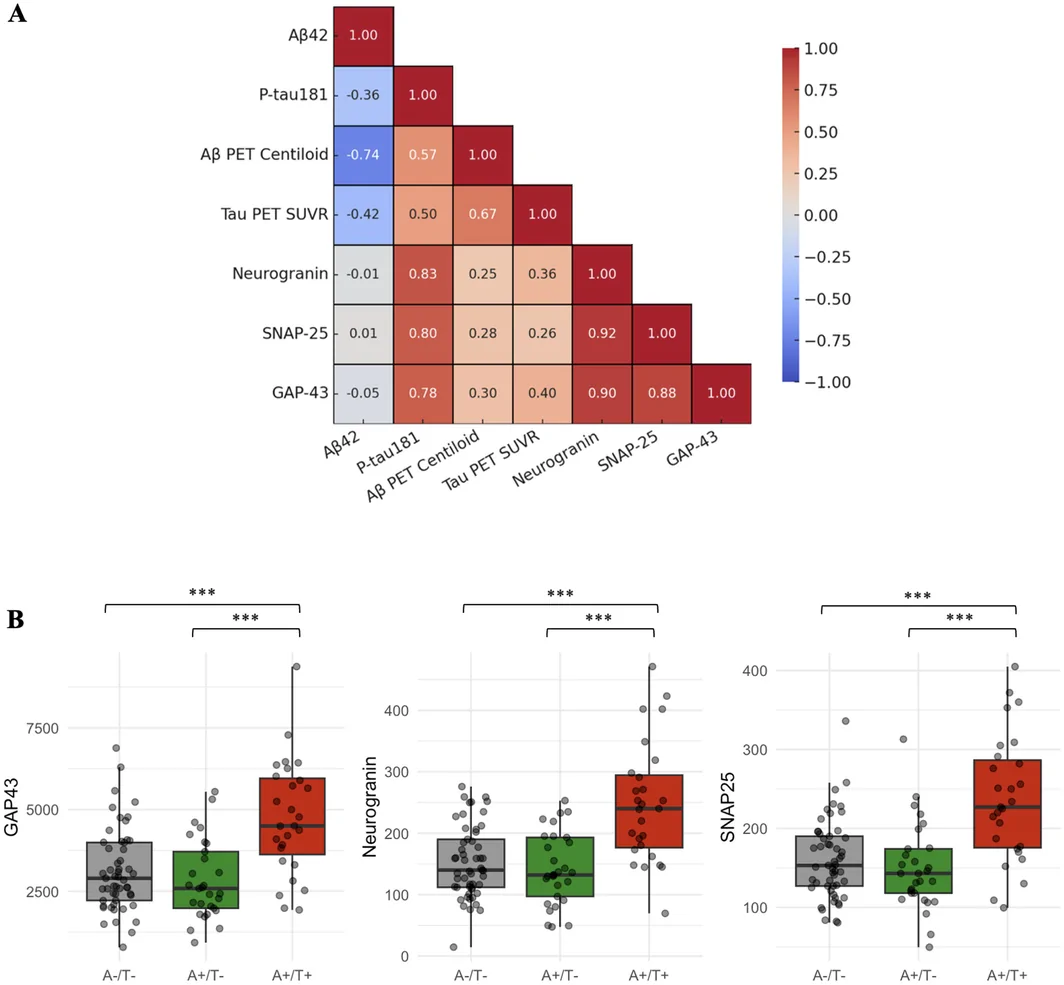
Associations and group differences of synaptic biomarkers in relation to amyloid and tau pathology (GMC cohort). (A) Heatmap displaying pairwise Spearman's rho correlations among CSF Aβ42, p‐tau181, Aβ PET Centiloid, Tau PET SUVR, and synaptic biomarkers (neurogranin, SNAP‐25, GAP‐43). Strongest correlations were observed between p‐tau181 and synaptic markers. (B) Boxplots illustrating the distribution of synaptic biomarker levels across study groups (A−/T−, A+/T−, and A+/T+). Synaptic biomarker levels were significantly elevated in the A+/T+ group compared to both the A−/T− and A+/T−, while no significant differences were observed between the A+/T− group and A−/T−. The horizontal lines within the boxplots represent the median values, while the boxes illustrate the interquartile range (IQR). Individual data points reflect the concentration of biomarker levels for each subject. *p < 0.05; **p < 0.01; ***p < 0.001.

#### 3.1.2 Replication Analyses in the ADNI Cohort

Consistent with the GMC cohort, GAP‐43 showed a strong correlation with CSF p‐tau181 (*ρ* = 0.76, *p* < 0.001), a moderate association with amyloid PET SUVR (*ρ* = 0.29, *p* < 0.001), and no correlation with CSF Aβ42 (*ρ* = −0.01, *p* = 0.758; Table S2). Scatterplots of GAP‐43 and Aβ PET SUVR or p‐tau181 are presented in Figure S2.

In the ADNI cohort, GAP‐43 levels differed across the A−/T− (*n* = 315), A+/T− (*n* = 171), A+/T+ (*n* = 256), and A−/T+ (*n* = 45) groups (*H(3)* = 287.9, *p* < 0.001). Specifically, GAP‐43 concentrations were higher in tau‐positive individuals, with comparable levels in the A+/T+ (7307 ± 3153 pg/mL) and A−/T+ (7536 ± 2784 pg/mL) groups, whereas lower levels were observed in tau‐negative groups, including A−/T− (4028 ± 1829 pg/mL) and A+/T− (3948 ± 1699 pg/mL).

### 3.2 Amyloid‐β and Tau Are Independently Associated With Synaptic Biomarkers

#### 3.2.1 GMC Cohort

Multiple linear regression analyses were performed to examine the associations between amyloid‐β and tau biomarkers and synaptic biomarker concentrations.

In the model including CSF Aβ42 and p‐tau181, both variables were associated with GAP‐43 (Aβ42: *β* = 0.31, 95% CI 0.23, 0.40, *p* < 0.001; p‐tau181: *β* = 0.94, 95% CI 0.84, 1.04, *p* < 0.001 for both comparisons). The model accounted for 81% of the variance in GAP‐43 (adj. *R* ^2^ = 0.805; Figure 2).

**FIGURE 2:**
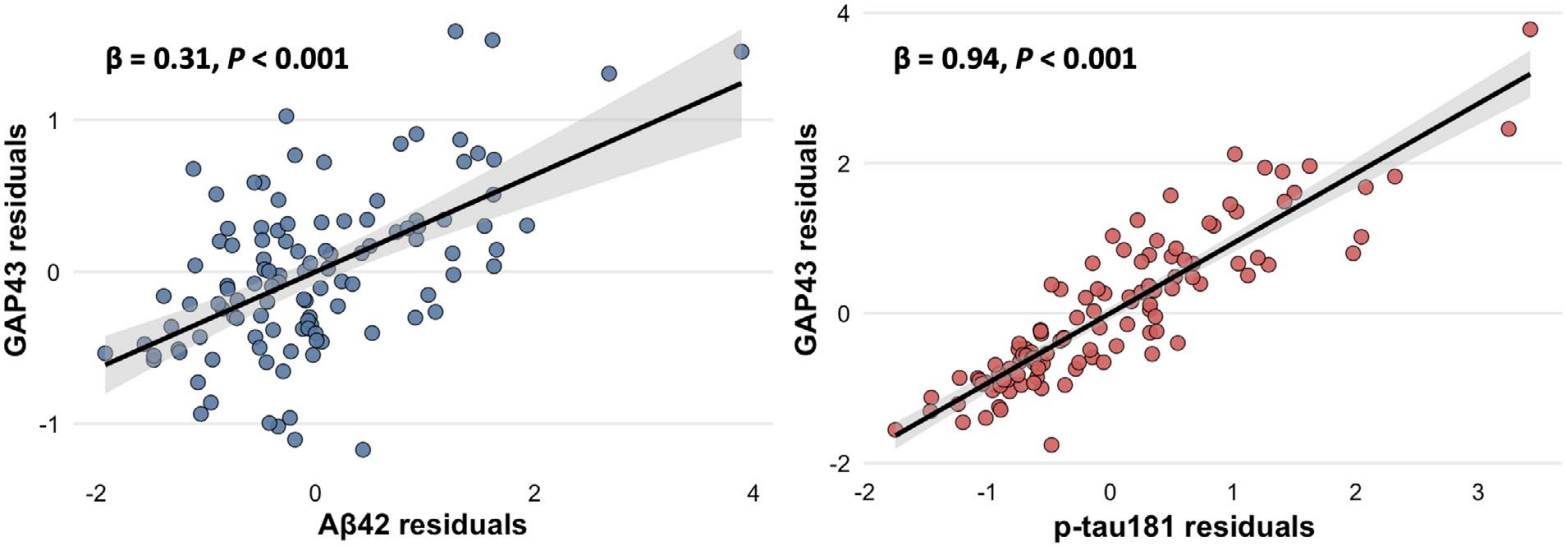
Associations of CSF Aβ42 and p‐tau181 with GAP‐43: Partial residual plots (GMC cohort). Partial residual plots illustrating independent associations of CSF Aβ42 (left) and p‐tau181 (right) with CSF GAP‐43 after controlling for the other biomarker. Higher Aβ42 and p‐tau181 levels are each associated with increased GAP‐43, with a stronger effect observed for p‐tau181, consistent with a predominant association of synaptic biomarkers with tau‐related biomarkers.

In the model including amyloid PET Centiloid values and CSF p‐tau181, higher CSF p‐tau181 values occurred with higher GAP‐43 concentrations (*β* = 1.02, 95% CI 0.86, 1.19, *p* < 0.001) whereas higher amyloid PET Centiloid values occurred with lower GAP‐43 concentrations (*β* = −0.31, 95% CI −0.46, −0.16, *p* < 0.001). This model explained 77% of the variance in GAP‐43 (adj. *R* ^2^ = 0.769; Figure 3).

**FIGURE 3:**
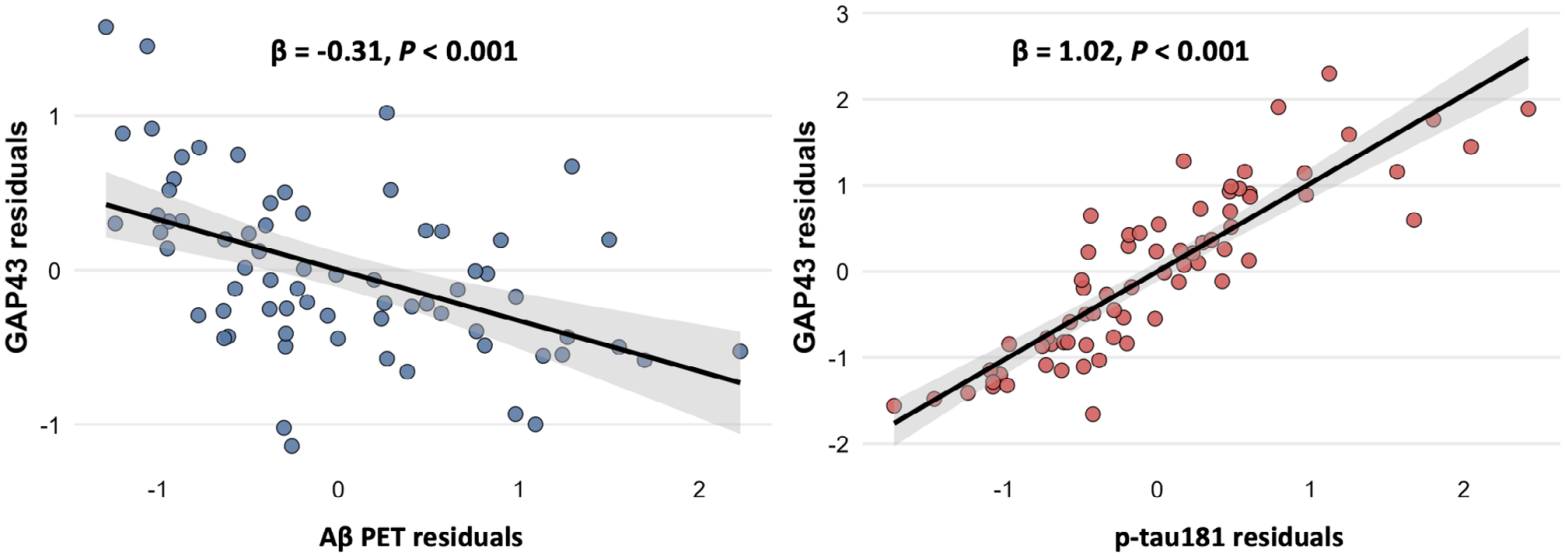
Associations of Aβ PET Centiloid and p‐tau181 with GAP‐43 (GMC cohort). Partial residual plots illustrating the independent associations of amyloid PET signal (left) and p‐tau181 (right) with CSF GAP‐43 after controlling for the other biomarker. Higher amyloid PET Centiloid is associated with lower GAP‐43, whereas p‐tau181 shows a strong positive association, consistent with a predominant association of synaptic biomarkers with tau‐related biomarkers.

Analyses of the additional synaptic biomarkers yielded comparable results. In models including CSF Aβ42 and CSF p‐tau181, higher concentrations of both biomarkers occurred with higher neurogranin (Aβ42: *β* = 0.25, 95% CI 0.15, 0.34; p‐tau181: *β* = 0.91, 95% CI: 0.80, 1.02; *adj. R* ^*2*^ = 0.749; *p* < 0.001) and higher SNAP‐25 concentrations (Aβ42: *β* = 0.32, 95% CI 0.22, 0.42; p‐tau181: *β* = 0.94, 95% CI: 0.84, 1.04; *adj. R* ^*2*^ = 0.814; *p* < 0.001).

Similarly, in models including amyloid PET Centiloid values and CSF p‐tau181, higher CSF p‐tau181 values occurred with higher neurogranin (*β* = 0.96, 95% CI 0.81, 1.10; *p* < 0.001; *adj. R* ^*2*^ = 0.753) and higher SNAP‐25 concentrations (*β* = 1.01, 95% CI 0.85, 1.16; *p* < 0.001; *adj. R* ^*2*^ = 0.782), whereas higher amyloid PET Centiloid values occurred with lower neurogranin (*β* = −0.16, 95% CI: −0.31, −0.02; *p* = 0.025) and SNAP‐25 concentrations (*β* = −0.25, 95% CI −0.38, −0.11; *p* < 0.001).

Regression models were repeated in the subset of participants with tau PET data. Within this subgroup (*n* = 48), 19 (39.6%) were A−/T−, 15 (31.3%) A+/T−, and 14 (29.2%) A+/T+.

In the model including CSF Aβ42 and tau PET global meta‐ROI, higher tau PET values occurred with higher GAP‐43 concentrations (*β* = 0.41, 95% CI 0.11, 0.72, *p* = 0.007), whereas no clear association was observed for CSF Aβ42 (*β* = 0.15, 95% CI −0.13, 0.43, *p* = 0.282; Figure S3).

In the model including both amyloid PET Centiloid and tau PET meta‐ROI, tau PET showed a trend toward association with GAP‐43 (*β* = 0.34, 95% CI: 0.00 to 0.69, *p* = 0.052), while amyloid PET Centiloid showed no clear association (*β* = 0.07, 95% CI: −0.29 to 0.43, *p* = 0.706; Figure S4).

#### 3.2.2 Replication Analyses in the ADNI Cohort

In the model including CSF Aβ42 and CSF p‐tau181, both biomarkers were associated with GAP‐43 (Aβ42: *β* = 0.17, 95% CI 0.12, 0.21; p‐tau181: *β* = 0.77, 95% CI 0.70, 0.84; *p* < 0.001 for both). The model explained 55% of the variance in GAP‐43 (adj. *R* ^2^ = 0.546).

In the model including amyloid PET SUVR and CSF p‐tau181, higher CSF p‐tau181 values occurred with higher GAP‐43 concentrations (*β* = 0.80, 95% CI 0.72, 0.87; *p* < 0.001), whereas higher amyloid PET SUVR values occurred with lower GAP‐43 concentrations (*β* = −0.14, 95% CI −0.21, −0.08; *p* < 0.001). This model explained 54% of the variance in GAP‐43 (adjusted *R* ^2^ = 0.536; Figure 4).

**FIGURE 4:**
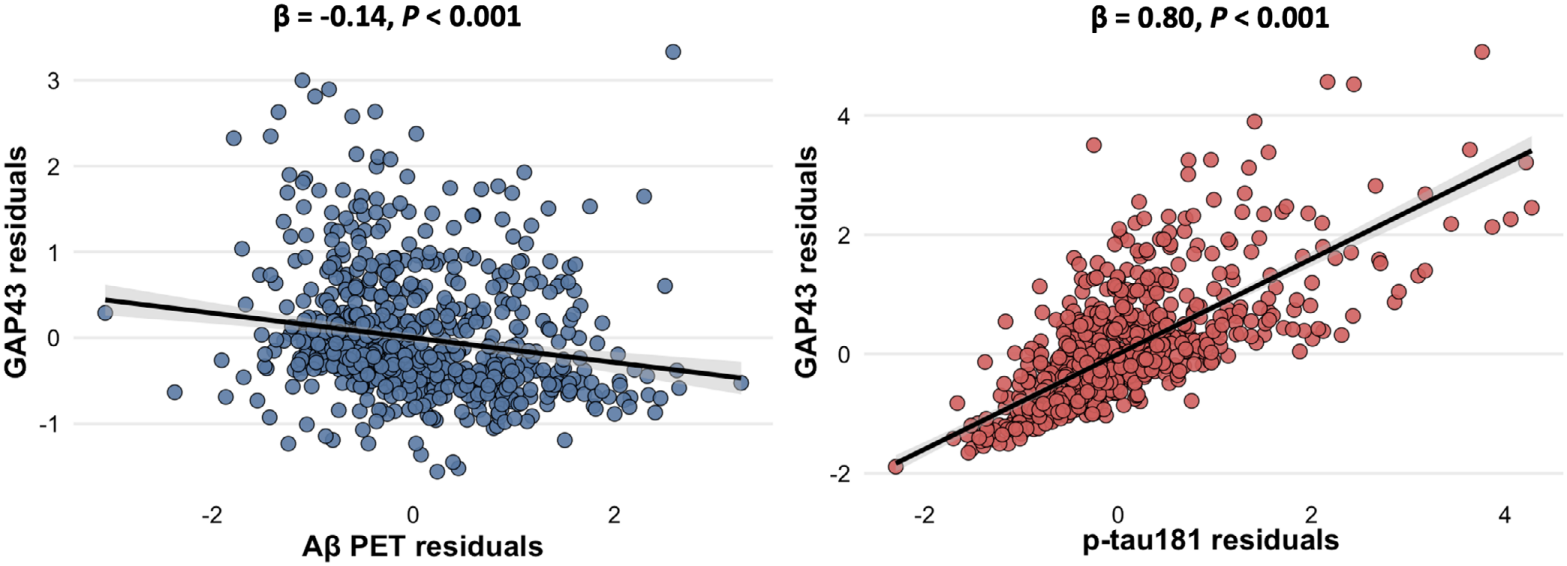
Associations of Aβ PET SUVR and p‐tau181 with GAP‐43 (ADNI cohort). Partial residual plots illustrating the independent associations of amyloid PET SUVR (left) and p‐tau181 (right) with CSF GAP‐43 after controlling for the other biomarker. Higher amyloid PET SUVR is associated with lower GAP‐43, whereas p‐tau181 shows a strong positive association, indicating a dominant tau‐related contribution to synaptic biomarker variation.

Sensitivity analyses incorporating additional demographic and clinical covariates and alternative tau measures yielded results consistent with the primary analyses. Details of all sensitivity analyses and model diagnostics, including VIF values showing no relevant multicollinearity among predictors, are reported in the Results and Tables S3–S11.

### 3.3 Tau Mediates the Association Between Amyloid‐β and Synaptic Damage

Mediation analyses examined whether the association between amyloid burden and GAP‐43 concentrations operated through CSF p‐tau181. In the GMC cohort (Figure 5A), the direct pathway from amyloid PET Centiloid to GAP‐43 was characterized by a negative coefficient (c' *β* = −0.328, *p* < 0.001), whereas the indirect pathway through CSF p‐tau181 showed a coefficient in the opposite direction (ab *β* = 0.586), resulting in a positive total effect (c *β* = 0.258), consistent with a suppression effect. A comparable suppression pattern was observed using CSF total tau as the mediator (Table S12).

**FIGURE 5:**
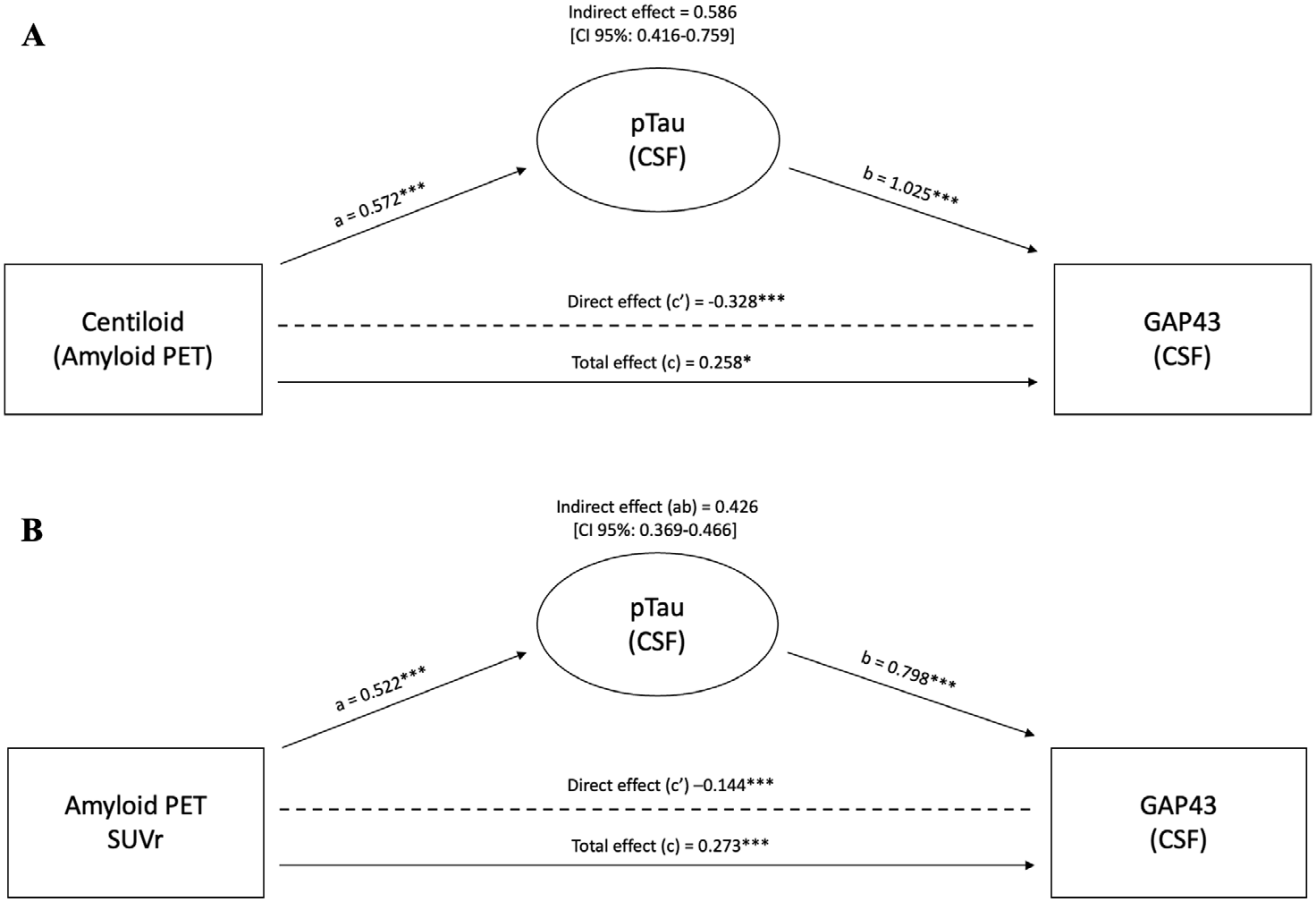
Mediation model of the association between amyloid burden and GAP‐43 via CSF p‐tau181 (GMC, ADNI). Mediation model depicting the indirect effect of Aβ PET Centiloid/SUVR on CSF GAP‐43 levels via p‐Tau181 in the GMC (A) or ADNI (B) cohort. The indirect association via p‐tau181 is opposite in sign to and larger than the direct association, consistent with statistical suppression. *p < 0.05; **p < 0.01; ***p < 0.001.

A comparable pattern was observed in the ADNI cohort (Figure 5B), where the direct pathway between amyloid PET SUVR and GAP‐43 showed a negative coefficient, while the indirect pathway through CSF p‐tau181 showed a coefficient in the opposite direction.

## 4 Discussion

In this study, we investigated the independent associations between amyloid‐β, tau, and synaptic damage across the Alzheimer's disease continuum. Leveraging two independent cohorts and complementary biomarker modalities, our findings indicate that synaptic biomarkers are predominantly associated with tau‐related biomarkers, while amyloid‐β could exert a more complex and indirect influence. These results may help refine current models of Alzheimer's disease pathophysiology by clarifying the relative associations of amyloid and tau with synaptic injury, one of the strongest biological correlates of cognitive decline [36].

Across two independent cohorts, synaptic biomarkers were increased in individuals with tau positivity, whereas amyloid positivity in the absence of tau pathology was not associated with substantial synaptic change. This model was particularly robust for GAP‐43, whose results were replicated in the large ADNI dataset, including the amyloid‐negative and tau‐positive group, which had synaptic biomarker levels comparable to those observed in individuals in the amyloid‐positive and tau‐positive group. These findings are consistent with a predominant role of tau‐related processes in synaptic injury, in line with neuropathological and observational studies showing tight spatial and quantitative relationships between tau aggregates, synapse loss, and cognitive decline [3, 23, 35, 37]. Consistent with our results, recent evidence from Lewy body disease indicates that CSF synaptic biomarkers such as SNAP‐25 and neurogranin are selectively increased in individuals with concomitant tau pathology, whereas amyloid positivity in the absence of tau does not translate into significant synaptic biomarker changes [38].

Correlation analyses further reinforced this interpretation. While synaptic biomarkers showed only weak‐to‐moderate associations with amyloid PET burden and no significant relationship with CSF Aβ42, they were strongly correlated with CSF p‐tau181 and, to a lesser extent, with tau PET SUVR. The stronger associations observed with CSF p‐tau181 compared with tau PET may reflect differences in biological meaning and temporal sensitivity, with p‐tau181 capturing soluble, phosphorylated tau species that increase early in response to amyloid pathology, whereas tau PET reflects more advanced, aggregated pathology [39, 40].

The regression analyses clarified the independent associations of amyloid‐β and tau biomarkers with synaptic biomarkers. Both CSF Aβ42 and p‐tau181 showed positive associations with synaptic biomarkers. When amyloid PET burden replaced CSF Aβ42, higher amyloid PET values occurred with lower synaptic biomarker concentrations after accounting for tau, whereas higher CSF p‐tau181 remained associated with higher synaptic biomarker concentrations. Because lower CSF Aβ42 and higher amyloid PET burden both index greater amyloid pathology, the positive Aβ42 coefficient and the negative amyloid PET coefficient are directionally concordant, indicating that greater amyloid burden is associated with lower synaptic biomarker concentrations once tau is accounted for. CSF Aβ42 is generally interpreted as reflecting early amyloid dysmetabolism and plaque formation, whereas amyloid PET primarily captures fibrillar plaque burden at later stages [41]. The inverse association between amyloid PET burden and synaptic biomarkers, once tau is taken into account, is consistent with the possibility that amyloid plaques themselves may not be directly synaptotoxic and may reflect a stage in which soluble, synaptotoxic Aβ species have been sequestered into less biologically active aggregates [42]. Importantly, the weaker associations of amyloid biomarkers should not be interpreted as evidence that amyloid pathology is unrelated to synaptic damage. Both CSF Aβ42 and amyloid PET tend to plateau once amyloid pathology is established, which reduces their variance at later disease stages and limits their ability to capture downstream processes.

Our findings are also consistent with recent multimodal PET evidence suggesting a reciprocal relationship between tau pathology and synaptic integrity [43]. Synapse‐rich brain regions appear to be preferentially vulnerable to tau accumulation, while increasing tau burden is associated with both local and network‐level synaptic loss. Together, these findings support the view that synaptic dysfunction is tightly coupled to tau‐related mechanisms throughout the course of Alzheimer's disease.

The interpretation of these findings also depends on how tau is operationalized in current biological frameworks. In the 2018 NIA‐AA research framework, CSF p‐tau181 was used as a biomarker of tau pathology (T) [44]. In the 2024 revised Alzheimer's Association criteria, soluble phosphorylated tau species, including CSF p‐tau181, are classified as Core 1 biomarkers that become abnormal early in response to amyloid pathology, whereas tau PET is a Core 2 biomarker reflecting aggregated tau and more advanced disease stages [45]. Consistently, kinetic and biomarker studies indicate that CSF phosphorylated tau increases with amyloid deposition, reflecting altered tau phosphorylation and neuronal secretion, before tau aggregates become detectable by PET [46, 47]. Accordingly, CSF p‐tau181 in our study, and the T status derived from it in participants without tau PET, should be regarded as an index of soluble tau‐related processes rather than as a direct measure of neurofibrillary tangle burden. From this perspective, the strong association between synaptic biomarkers and CSF p‐tau181 may partly reflect a common amyloid‐related neuronal response, whereas the more modest associations with tau PET, observed in a smaller subgroup, suggest that aggregated tau explains a smaller proportion of the variance in CSF synaptic biomarkers. Similarly, the high GAP‐43 levels observed in the ADNI A−/T+ group should be interpreted with caution: a CSF A−/T+ profile is uncommon and has been associated with a benign clinical course and with high CSF Aβ concentrations [48].

Mediation analyses provided convergent, although cross‐sectional, support for this interpretation, showing a negative direct association between amyloid burden and synaptic biomarkers, but a stronger positive indirect association through p‐tau181, resulting in an overall positive association. This pattern is consistent with a model in which amyloid‐β contributes to synaptic alterations largely through its relationship with tau, rather than through a direct effect on synapses. Tau therefore appears more closely coupled to synaptic injury in vivo, in line with experimental work indicating that tau pathology is linked to synaptic dysfunction and degeneration [19].

These findings help reconcile previous studies that have reported differing relationships between amyloid, tau and synaptic biomarkers [10, 21, 38]. Our biomarker‐based approach that distinguishes between amyloid and tau contributions may therefore help clarify the pathophysiological sequence linking Aβ, tau and synaptic dysfunction.

Previous studies reported heterogeneous synaptic biomarker levels in A+/T− individuals, ranging from increased [21] to reduced [49] concentrations compared with biomarker‐negative controls, whereas increases have consistently been observed once tau‐related changes emerge [49]. In our cohorts, A+/T− individuals had synaptic biomarker levels comparable to those of A−/T− individuals. Other presynaptic proteins, such as β‐synuclein, are increased already at the preclinical stage of AD [50], supporting the use of multimarker panels reflecting different synaptic compartments. Beyond pathophysiology, these findings have potential clinical implications. Synaptic biomarkers have shown prognostic value for cognitive decline, particularly in amyloid‐positive individuals [7, 14], and may therefore contribute to the stratification of patients at risk of progression. Their clinical utility would be maximized by reliable measurement in blood: plasma SNAP‐25 correlates with its CSF counterpart [51], and serum β‐synuclein increases along the AD continuum and is associated with cognitive decline [52]. Moreover, CSF neurogranin has been included among downstream biomarkers in anti‐amyloid trials [53], suggesting that synaptic biomarkers could help monitor the downstream effects of disease‐modifying therapies [18]. Our results indicate that such changes should be interpreted in light of the close association of synaptic biomarkers with tau‐related processes. Future longitudinal studies, stratified by clinical stage and integrating cognitive outcomes and blood‐based synaptic biomarkers, are needed to establish their prognostic and monitoring value.

Several strengths should be acknowledged, including the use of multiple synaptic biomarkers, multimodal assessment of amyloid and tau pathology, robust statistical modeling and replication in a large independent cohort. Limitations should also be considered. The cross‐sectional design limits inference on temporal ordering, and longitudinal studies would be required to better characterize the sequence of changes in amyloid, tau and synaptic biomarkers. The number of participants with tau PET was modest; moreover, some degree of overfitting cannot be excluded in the adjusted models in the PET subsets. Furthermore, because p‐tau181 and the synaptic proteins are all neuronally derived and released into cerebrospinal fluid, part of their strong association could reflect shared neuronal secretion and CSF dynamics rather than a tau‐specific effect on synapses. This possibility cannot be excluded with the present data: CSF t‐tau has itself been proposed as a closer proxy of synaptic than of axonal degeneration [54], so that the convergent results obtained with t‐tau support the robustness of our findings but do not disentangle tau‐specific from more general neurodegenerative processes. Markers of neuroaxonal injury, such as NfL, would help to address this issue. When analyses were restricted to amyloid‐positive participants, CSF p‐tau181 remained strongly associated with GAP‐43, with effect sizes comparable to the full sample, whereas amyloid PET was no longer significant. This indicates that the p‐tau181–GAP‐43 association was not solely driven by the A−/A+ contrast. Further limitations include the availability of PET imaging only in a subset of GMC participants, potentially introducing selection bias, and the use of visual PET reads for A/T classification, although quantitative PET measures were used in the analyses. No A−/T+ individuals were identified in GMC, likely due to the limited sample size, whereas their prevalence in ADNI (5.7%) was consistent with previous estimates [48]. Information on common copathologies, particularly α‐synuclein, was not available: concomitant α‐synuclein pathology has been associated with lower CSF neurogranin, p‐tau181 and t‐tau levels in autopsy‐confirmed AD [55], whereas CSF and plasma SNAP‐25 appeared unaffected by α‐synuclein seed amplification assay positivity [51], so that undetected mixed pathology may have contributed to the variability of some synaptic biomarkers.

In summary, these results have implications for the understanding of biomarker‐based models of Alzheimer's disease and for the interpretation of synaptic biomarkers in research and clinical settings. The findings suggest that synaptic biomarkers are primarily associated with tau‐related processes and may serve as indicators of tau‐related neuronal injury. They are further consistent with a framework in which amyloid‐β contributes to synaptic alterations predominantly through tau‐related mechanisms.

## Author Contributions

F.R.(Roveta), F.R.(Ribaldi), and G.B.F. contributed to the conception and design of the study. F.R.(Roveta), A.J.M., V.G., C.B., A.L., N.J.A., H.Z., F.R.(Ribaldi), and G.B.F. contributed to the acquisition and analysis of data. F.R.(Roveta), I.R., E.R., F.R.(Ribaldi), and G.B.F. contributed to drafting the text or preparing the figures.

## Funding

The projects of the Centre de la mémoire are funded by the State of Geneva and the following private donors under the supervision of the Private Foundation of Geneva University Hospitals: A.P.R.A.—Association Suisse pour la Recherche sur la Maladie d'Alzheimer, Genève; Fondation Segré, Genève; Race Against Dementia Foundation, London, UK; Fondation Child Care, Genève; Fondation Edmond J. Safra, Genève; Fondation Minkoff, Genève; Fondazione Agusta, Lugano; McCall Macbain Foundation, Canada; Nicole et René Keller, Genève; Fondation AETAS, Genève. Associazione Italiana Ricerca Alzheimer Onlus (AirAlzh‐AGYR‐2023); Italian Ministry of the University (MUR), projects “Dipartimenti di Eccellenza 2023–2027”, to the Department of Neuroscience “Rita Levi‐Montalcini”, University of Torino, Italy. Federica Ribaldi is supported by the Swiss National Science Foundation (320030_182772 and 32003B0_220269). Henrik Zetterberg is a Wallenberg Scholar and a Distinguished Professor at the Swedish Research Council supported by grants from the Swedish Research Council (#2023‐00356, #2022‐01018 and #2019‐02397), the European Union's Horizon Europe research and innovation programme under grant agreement No 101053962, and Swedish State Support for Clinical Research (#ALFGBG‐71320).

## Consent

The study was approved by the Regional Research Ethics Committee (PB_2016‐01346, 2020_00403) and conducted in accordance with the principles of the Declaration of Helsinki and the International Conference on Harmonization Good Clinical Practice. All participants gave their written informed consent to participate in this study.

## Conflicts of Interest

Fausto Roveta, Augusto J. Mendes, Valentina Garibotto, Cecilia Boccalini, Aurelien Lathuiliere, Innocenzo Rainero, Elisa Rubino, Nicholas J. Ashton, Henrik Zetterberg, Federica Ribaldi, and Giovanni B. Frisoni declare no conflicts of interest in relation to this study.

## Supporting information


**Table S1:** Demographics, cognitive stages, and biomarkers status of subjects by study groups (ADNI).
**Table S2:** Spearman's correlations among CSF and PET biomarkers.
**Table S3:** Linear multiple regression, dependent variable: GAP43 (N = 98, GMC).
**Table S4:** Linear multiple regression, dependent variable: GAP43 (N = 787, ADNI).
**Table S5:** Linear multiple regression, dependent variable: GAP43 (N = 62, GMC).
**Table S6:** Linear multiple regression model, dependent variable: GAP43 (N = 777, ADNI).
**Table S7:** Multicollinearity and goodness‐of‐fit diagnostics for the primary regression models predicting synaptic biomarker concentrations.
**Table S8:** Linear multiple regression, dependent variable: GAP43 (N = 98, GMC).
**Table S9:** Linear multiple regression, dependent variable: GAP43 (N = 62, GMC).
**Table S10:** Sensitivity analysis including global cognitive performance (MMSE) in the adjusted linear regression model (N = 109, GMC cohort).
**Table S11:** Sensitivity analysis restricted to amyloid‐positive participants (ADNI cohort).
**Table S12:** Mediation analysis including total tau as mediator of the relationship between amyloid and GAP43 (N = 69, GMC).
**Figure S1:** Scatterplot of GAP43 versus Aβ PET Centiloid and pTau181 (GMC cohort).
**Figure S2:** Scatterplot of GAP43 versus Amyloid PET SUVR and pTau181 (ADNI cohort).
**Figure S3:** Associations of CSF Aβ42 and tau PET global meta‐ROI with GAP‐43: partial residual plots (GMC cohort).
**Figure S4:** Associations of amyloid PET and Tau PET global meta‐ROI with GAP43: partial residual plots (GMC cohort).

**Data S1:** ADNI_Acknowledgement_List.

## Data Availability

The data that support the findings of this study are available on request from the corresponding author. The data are not publicly available due to privacy or ethical restrictions.

## References

[1] G. B. Frisoni , O. Hansson , E. Nichols , et al., “New Landscape of the Diagnosis of Alzheimer's Disease,” Lancet 406 (2025): 1389–1407, 10.1016/S0140-6736(25)01294-2. 40997838

[2] C. Tristão‐Pereira , S. Langella , A. Baena , et al., “Contribution of Local Amyloid‐β and Tau Burden to Hypometabolism in Autosomal‐Dominant Alzheimer's Disease,” Brain Communications 8, no. 1 (2026): fcaf508, 10.1093/braincomms/fcaf508. PMC12816921 41567829

[3] R. D. Terry , E. Masliah , D. P. Salmon , et al., “Physical Basis of Cognitive Alterations in Alzheimer's Disease: Synapse Loss Is the Major Correlate of Cognitive Impairment,” Annals of Neurology 30, no. 4 (1991): 572–580, 10.1002/ana.410300410. 1789684

[4] C. Boccalini , F. Ribaldi , I. Hristovska , et al., “The Impact of Tau Deposition and Hypometabolism on Cognitive Impairment and Longitudinal Cognitive Decline,” Alzheimer's & Dementia 20, no. 1 (2024): 221–233, 10.1002/alz.13355. PMC10916991 37555516

[5] F. Roveta , A. Cermelli , S. Boschi , et al., “Synaptic Proteins as Fluid Biomarkers in Alzheimer's Disease: A Systematic Review and Meta‐Analysis,” Journal of Alzheimer's Disease 90, no. 4 (2022): 1381–1393, 10.3233/JAD-220515. 36278349

[6] M. C. De Wilde , C. R. Overk , J. W. Sijben , and E. Masliah , “Meta‐Analysis of Synaptic Pathology in Alzheimer's Disease Reveals Selective Molecular Vesicular Machinery Vulnerability,” Alzheimer's & Dementia 12, no. 6 (2016): 633–644, 10.1016/j.jalz.2015.12.005. PMC5058345 26776762

[7] R. Tarawneh , G. D'Angelo , D. Crimmins , et al., “Diagnostic and Prognostic Utility of the Synaptic Marker Neurogranin in Alzheimer Disease,” JAMA Neurology 73, no. 5 (2016): 561, 10.1001/jamaneurol.2016.0086. PMC4861689 27018940

[8] A. Brinkmalm , G. Brinkmalm , W. G. Honer , et al., “SNAP‐25 Is a Promising Novel Cerebrospinal Fluid Biomarker for Synapse Degeneration in Alzheimer's Disease,” Molecular Neurodegeneration 9, no. 1 (2014): 53, 10.1186/1750-1326-9-53. PMC4253625 25418885

[9] Å. Sandelius , E. Portelius , Å. Källén , et al., “Elevated CSF GAP‐43 Is Alzheimer's Disease Specific and Associated With Tau and Amyloid Pathology,” Alzheimer's & Dementia 15, no. 1 (2019): 55–64, 10.1016/j.jalz.2018.08.006. PMC6333489 30321501

[10] M. Milà‐Alomà , A. Brinkmalm , N. J. Ashton , et al., “CSF Synaptic Biomarkers in the Preclinical Stage of Alzheimer Disease and Their Association With MRI and PET: A Cross‐Sectional Study,” Neurology 97, no. 21 (2021): e2065–e2078, 10.1212/WNL.0000000000012853. PMC8610620 34556565

[11] Y. T. Wang , N. J. Ashton , S. Servaes , et al., “The Relation of Synaptic Biomarkers With Aβ, Tau, Glial Activation, and Neurodegeneration in Alzheimer's Disease,” Translational Neurodegeneration 13, no. 1 (2024): 27, 10.1186/s40035-024-00420-1. PMC11131272 38802928

[12] W. Gulisano , M. Melone , C. Ripoli , et al., “Neuromodulatory Action of Picomolar Extracellular Aβ42 Oligomers on Presynaptic and Postsynaptic Mechanisms Underlying Synaptic Function and Memory,” Journal of Neuroscience 39, no. 30 (2019): 5986–6000, 10.1523/JNEUROSCI.0163-19.2019. PMC6650983 31127002

[13] F. Fagiani , C. Lanni , M. Racchi , A. Pascale , and S. Govoni , “Amyloid‐β and Synaptic Vesicle Dynamics: A Cacophonic Orchestra,” Journal of Alzheimer's Disease 72, no. 1 (2019): 1–14, 10.3233/JAD-190771. 31561377

[14] A. Öhrfelt , A. L. Benedet , N. J. Ashton , et al., “Association of CSF GAP‐43 With the Rate of Cognitive Decline and Progression to Dementia in Amyloid‐Positive Individuals,” Neurology 100, no. 3 (2023): e275–e285, 10.1212/WNL.0000000000201417. PMC9869758 36192174

[15] R. Ellingford , S. S. Harris , M. Kehring , et al., “Alzheimer's Disease Pathology Degrades an NMDA Receptor‐Dependent Spontaneous Activity Pattern in Cortico‐Hippocampal Circuits,” Neuron 114, no. 13 (2026): 2386–2403.e9, 10.1016/j.neuron.2026.02.027. PMC13045655 41916281

[16] Q. Qiang , L. Skudder‐Hill , T. Toyota , W. Wei , and H. Adachi , “CSF GAP‐43 as a Biomarker of Synaptic Dysfunction Is Associated With Tau Pathology in Alzheimer's Disease,” Scientific Reports 12, no. 1 (2022): 17392, 10.1038/s41598-022-20324-2. PMC9576773 36253408

[17] S. H. Wolner , H. S. Gleerup , C. S. Musaeus , et al., “Synaptosomal‐Associated Protein 25 kDA (SNAP‐25) Levels in Cerebrospinal Fluid: Implications for Alzheimer's Disease Diagnosis and Monitoring,” Synapse 79, no. 2 (2025): e70010, 10.1002/syn.70010. PMC11800177 39912369

[18] J. L. Cummings , C. E. Teunissen , B. K. Fiske , et al., “Biomarker‐Guided Decision Making in Clinical Drug Development for Neurodegenerative Disorders,” Nature Reviews Drug Discovery 24, no. 8 (2025): 589–609, 10.1038/s41573-025-01165-w. 40185982

[19] N. Franzmeier , A. Dehsarvi , A. Steward , et al., “Elevated CSF GAP‐43 Is Associated With Accelerated Tau Accumulation and Spread in Alzheimer's Disease,” Nature Communications 15, no. 1 (2024): 202, 10.1038/s41467-023-44374-w. PMC10764818 38172114

[20] J. W. Wu , S. A. Hussaini , I. M. Bastille , et al., “Neuronal Activity Enhances Tau Propagation and Tau Pathology in Vivo,” Nature Neuroscience 19, no. 8 (2016): 1085–1092, 10.1038/nn.4328. PMC4961585 27322420

[21] J. B. Pereira , S. Janelidze , R. Ossenkoppele , et al., “Untangling the Association of Amyloid‐β and Tau With Synaptic and Axonal Loss in Alzheimer's Disease,” Brain 144, no. 1 (2021): 310–324, 10.1093/brain/awaa395. PMC8210638 33279949

[22] G. B. Frisoni , D. Altomare , D. R. Thal , et al., “The Probabilistic Model of Alzheimer Disease: The Amyloid Hypothesis Revised,” Nature Reviews Neuroscience 23, no. 1 (2022): 53–66, 10.1038/s41583-021-00533-w. PMC8840505 34815562

[23] A. Moscoso , F. Heeman , S. Raghavan , et al., “Frequency and Clinical Outcomes Associated With Tau Positron Emission Tomography Positivity,” Journal of the American Medical Association 334, no. 3 (2025): 229–242, 10.1001/jama.2025.7817. PMC12848857 40522652

[24] F. Ribaldi , C. Chicherio , D. Altomare , et al., “Brain Connectivity and Metacognition in Persons With Subjective Cognitive Decline (COSCODE): Rationale and Study Design,” Alzheimer's Research & Therapy 13, no. 1 (2021): 105, 10.1186/s13195-021-00846-z. PMC8152092 34034799

[25] USC Stevens Neuroimaging and Informatics Institute , “Image & Data Archive,” accessed November 1, 2024, ida.loni.usc.edu.

[26] D. E. Peretti , C. Boccalini , F. Ribaldi , et al., “Association of Glial Fibrillary Acid Protein, Alzheimer's Disease Pathology and Cognitive Decline,” Brain 147, no. 12 (2024): 4094–4104, 10.1093/brain/awae211. PMC11629700 38940331

[27] M. Navitsky , A. D. Joshi , I. Kennedy , et al., “Standardization of Amyloid Quantitation With Florbetapir Standardized Uptake Value Ratios to the Centiloid Scale,” Alzheimer's & Dementia 14, no. 12 (2018): 1565–1571, 10.1016/j.jalz.2018.06.1353. 30006100

[28] A. J. Schwarz , S. Shcherbinin , L. J. Slieker , et al., “Topographic Staging of Tau Positron Emission Tomography Images,” Alzheimer's & Dementia: Diagnosis, Assessment & Disease Monitoring 10, no. 1 (2018): 221–231, 10.1016/j.dadm.2018.01.006. PMC5956800 29780867

[29] S. Mishra , B. A. Gordon , Y. Su , et al., “AV‐1451 PET Imaging of Tau Pathology in Preclinical Alzheimer Disease: Defining a Summary Measure,” NeuroImage 161 (2017): 171–178, 10.1016/j.neuroimage.2017.07.050. PMC5696044 28756238

[30] European Medicines Agency , “Homepage,” accessed December 1, 2024, https://www.ema.europa.eu/en/homepage.

[31] A. S. Fleisher , M. J. Pontecorvo , M. D. Devous , et al., “Positron Emission Tomography Imaging With [^18^ F]Flortaucipir and Postmortem Assessment of Alzheimer Disease Neuropathologic Changes,” JAMA Neurology 77, no. 7 (2020): 829, 10.1001/jamaneurol.2020.0528. PMC7186920 32338734

[32] J. Therriault , T. A. Pascoal , F. Z. Lussier , et al., “Biomarker Modeling of Alzheimer's Disease Using PET‐Based Braak Staging,” Nature Aging 2, no. 6 (2022): 526–535, 10.1038/s43587-022-00204-0. PMC10154209 37118445

[33] A. J. Mendes , F. Ribaldi , A. Lathuiliere , et al., “Head‐To‐Head Study of Diagnostic Accuracy of Plasma and Cerebrospinal Fluid p‐tau217 Versus p‐tau181 and p‐tau231 in a Memory Clinic Cohort,” Journal of Neurology 271, no. 4 (2024): 2053–2066, 10.1007/s00415-023-12148-5. PMC10972950 38195896

[34] A. D. Joshi , M. J. Pontecorvo , C. M. Clark , et al., “Performance Characteristics of Amyloid PET With Florbetapir F 18 in Patients With Alzheimer's Disease and Cognitively Normal Subjects,” Journal of Nuclear Medicine 53, no. 3 (2012): 378–384, 10.2967/jnumed.111.090340. 22331215

[35] M. Bucci , K. Chiotis , A. Nordberg , and for the Alzheimer's Disease Neuroimaging Initiative , “Alzheimer's Disease Profiled by Fluid and Imaging Markers: Tau PET Best Predicts Cognitive Decline,” Molecular Psychiatry 26, no. 10 (2021): 5888–5898, 10.1038/s41380-021-01263-2. PMC8758489 34593971

[36] S. Lista , A. Santos‐Lozano , E. Emanuele , et al., “Monitoring Synaptic Pathology in Alzheimer's Disease Through Fluid and PET Imaging Biomarkers: A Comprehensive Review and Future Perspectives,” Molecular Psychiatry 29, no. 3 (2024): 847–857, 10.1038/s41380-023-02376-6. 38228892

[37] G. Vanderlinden , M. Koole , L. Michiels , R. Lemmens , M. Vandenbulcke , and K. Van Laere , “Longitudinal Synaptic Loss Versus Tau Braak Staging in Amnestic Mild Cognitive Impairment,” Alzheimer's & Dementia 21, no. 2 (2025): e14412, 10.1002/alz.14412. PMC11848342 39732507

[38] L. Barba , S. Abu‐Rumeileh , S. Halbgebauer , et al., “CSF Synaptic Biomarkers in AT(N)‐Based Subgroups of Lewy Body Disease,” Neurology 101, no. 1 (2023): e50–e62, 10.1212/WNL.0000000000207371. PMC10351307 37188538

[39] R. Ossenkoppele , J. Reimand , R. Smith , et al., “Tau PET Correlates With Different Alzheimer's Disease‐Related Features Compared to CSF and Plasma p‐Tau Biomarkers,” EMBO Molecular Medicine 13, no. 8 (2021): e14398, 10.15252/emmm.202114398. PMC8350902 34254442

[40] N. Watamura , M. S. Foiani , S. Bez , et al., “In Vivo Hyperphosphorylation of Tau Is Associated With Synaptic Loss and Behavioral Abnormalities in the Absence of Tau Seeds,” Nature Neuroscience 28, no. 2 (2025): 293–307, 10.1038/s41593-024-01829-7. PMC11802456 39719507

[41] A. Leuzy , A. Bollack , D. Pellegrino , et al., “Considerations in the Clinical Use of Amyloid PET and CSF Biomarkers for Alzheimer's Disease,” Alzheimer's & Dementia 21, no. 3 (2025): e14528, 10.1002/alz.14528. PMC11881640 40042435

[42] T. Yang , S. Li , H. Xu , D. M. Walsh , and D. J. Selkoe , “Large Soluble Oligomers of Amyloid β‐Protein From Alzheimer Brain Are Far Less Neuroactive Than the Smaller Oligomers to Which They Dissociate,” Journal of Neuroscience 37, no. 1 (2017): 152–163, 10.1523/JNEUROSCI.1698-16.2016. PMC5214627 28053038

[43] Y. Luan , Q. Huang , J. Wang , et al., “Synaptic Density and Tau Pathology in Alzheimer's Disease: A Dual Role in Susceptibility and Degeneration,” Brain 149, no. 6 (2026): 1958–1972, 10.1093/brain/awaf425. 41231728

[44] C. R. Jack, Jr. , D. A. Bennett , K. Blennow , et al., “NIA‐AA Research Framework: Toward a Biological Definition of Alzheimer's Disease,” Alzheimer's & Dementia 14, no. 4 (2018): 535–562, 10.1016/j.jalz.2018.02.018. PMC5958625 29653606

[45] C. R. Jack, Jr. , J. S. Andrews , T. G. Beach , et al., “Revised Criteria for Diagnosis and Staging of Alzheimer's Disease: Alzheimer's Association Workgroup,” Alzheimer's & Dementia 20, no. 8 (2024): 5143–5169, 10.1002/alz.13859. PMC11350039 38934362

[46] C. Sato , N. R. Barthélemy , K. G. Mawuenyega , et al., “Tau Kinetics in Neurons and the Human Central Nervous System,” Neuron 97, no. 6 (2018): 1284–1298.e7, 10.1016/j.neuron.2018.02.015. PMC6137722 29566794

[47] N. Mattsson‐Carlgren , E. Andersson , S. Janelidze , et al., “Aβ Deposition Is Associated With Increases in Soluble and Phosphorylated Tau That Precede a Positive Tau PET in Alzheimer's Disease,” Science Advances 6, no. 16 (2020): eaaz2387, 10.1126/sciadv.aaz2387. PMC7159908 32426454

[48] P. Erickson , J. Simrén , W. S. Brum , et al., “Prevalence and Clinical Implications of a β‐Amyloid–Negative, Tau‐Positive Cerebrospinal Fluid Biomarker Profile in Alzheimer Disease,” JAMA Neurology 80, no. 9 (2023): 969–979, 10.1001/jamaneurol.2023.2338. PMC10391361 37523162

[49] J. Goossens , A. Cervantes González , N. Dewit , et al., “Evaluation of Cerebrospinal Fluid Levels of Synaptic Vesicle Protein, VAMP‐2, Across the Sporadic Alzheimer's Disease Continuum,” Alzheimer's Research & Therapy 15, no. 1 (2023): 186, 10.1186/s13195-023-01336-0. PMC10612328 37898760

[50] L. Barba , S. Abu Rumeileh , G. Bellomo , et al., “Cerebrospinal Fluid β‐Synuclein as a Synaptic Biomarker for Preclinical Alzheimer's Disease,” Journal of Neurology, Neurosurgery, and Psychiatry 94, no. 1 (2023): 83–86, 10.1136/jnnp-2022-329124. 35944974

[51] L. Gaetani , G. Bellomo , D. Chiasserini , et al., “Influence of Co‐Pathology on CSF and Plasma Synaptic Markers SNAP25 and VAMP2 in Alzheimer's Disease and Parkinson's Disease,” Alzheimer's Research & Therapy 17, no. 1 (2025): 115, 10.1186/s13195-025-01762-2. PMC12096548 40405270

[52] L. Barba , G. Bellomo , D. Alcolea , et al., “Serum Level Changes of the Synaptic Marker Beta‐Synuclein in Alzheimer's Disease Continuum and Other Dementias,” Journal of Neurology, Neurosurgery, and Psychiatry 96, no. 12 (2025): 1144–1153, 10.1136/jnnp-2025-336189. 40571405

[53] C. H. van Dyck , C. J. Swanson , P. Aisen , et al., “Lecanemab in Early Alzheimer's Disease,” New England Journal of Medicine 388, no. 1 (2023): 9–21, 10.1056/NEJMoa2212948. 36449413

[54] C. Soares , B. Bellaver , P. C. L. Ferreira , et al., “CSF Total Tau as a Proxy of Synaptic Degeneration,” Nature Communications 16, no. 1 (2025): 8076, 10.1038/s41467-025-63545-5. PMC12397218 40883301

[55] K. A. Q. Cousins , S. Arezoumandan , S. Shellikeri , et al., “CSF Biomarkers of Alzheimer Disease in Patients With Concomitant α‐Synuclein Pathology,” Neurology 99, no. 20 (2022): e2303–e2312, 10.1212/WNL.0000000000201202. PMC9694837 36041863

