# Supplementary material for: The Association of Amyloid‐β and Tau With Synaptic Dysfunction in Alzheimer's Disease
Source: Ann Clin Transl Neurol. 2026 Oct 30:10.1002/acn3.70558. Online ahead of print. doi: 10.1002/acn3.70558 (PMC13720864; doi:10.1002/acn3.70558)
Supplement: Supplementary file 1 [file ACN3-9999-0-s001.pdf]

The association of amyloid-β and tau with synaptic dysfunction in Alzheimer's disease

Fausto Roveta et al.

SUPPLEMENTARY MATERIALS

Supplementary Results

Across both cohorts, sensitivity analyses additionally adjusting for age, education, and APOE ε4 status yielded highly consistent results, with CSF p-tau181 remaining the strongest independent correlate of GAP43 and amyloid measures retaining their respective associations (Supplementary Tables 3–6).

VIF values were low across all primary models, indicating no relevant multicollinearity between amyloid and p-tau181 predictors; full model diagnostics are reported in Supplementary Table 7. In the GMC cohort, results were also consistent when using CSF t-tau instead of p-tau181 as the tau measure (Supplementary Tables 8–9), and when additionally adjusting for global cognitive performance (MMSE) (Supplementary Table 10). In this model, MMSE showed a modest positive conditional association with GAP-43 ($\beta = 0.12$), whereas the coefficients of CSF Aβ42 and p-tau181 were essentially unchanged.

Additional sensitivity analyses were repeated with primary regression models in the ADNI cohort restricted to amyloid-positive participants only (A+/T− and A+/T+, N=427), thereby removing the amyloid-negative contrast. CSF p-tau181 remained strongly associated with GAP-43 in both the CSF model (β=0.71, 95% CI 0.63–0.80, $P<0.001$; adj. R^2=0.519) and the amyloid PET model (β=0.73, 95% CI 0.65–0.82, $P<0.001$; adj. R^2=0.510), with magnitudes comparable to the full-sample analyses. In this amyloid-positive subgroup, amyloid PET SUVR was no longer significantly associated with GAP-43 (β=−0.08, 95% CI −0.17 to 0.01, P=0.085), while CSF Aβ42 retained a modest independent association (β=0.12, 95% CI 0.06–0.18, $P<0.001$) (Supplementary Table 11).

Supplementary Tables

Supplementary Table 1. Demographics, cognitive stages, and biomarkers status of subjects by study groups (ADNI).

Variable	**A-/T- (n = 315)**	**A+/T- (n = 171)**	**A+/T+ (n = 256)**	**A-/T+ (n = 45)**	**p-value**
**Demographics**					
Age (years) (Mean ± SD)	70 ± 7 [c]	73 ± 7 [b]	74 ± 7 [a]	75 ± 7 [a]	**< 0.001**
Education (years) (Mean ± SD)	17 ± 2 [a]	16 ± 3	16 ± 3 [b]	17 ± 2	**0.016**
Gender, female (%)	45	46	50	51	0.523
**Cognitive Stages and Global Scores**					
CU / MCI / Dementia (%)	44 / 52 / 3 [a]	29 / 53 / 18 [b]	14 / 55 / 31[c]	49 / 38 / 13 [a]	**< 0.001**
MMSE (Mean ± SD)	28.7 ± 1.6 [a]	27.6 ± 2.6 [a]	26.3 ± 2.8 [b]	27.9 ± 2.6 [a]	**< 0.001**
**PET imaging**					
Amyloid PET SUVR (Mean ± SD)	1.011 ± 0.053 [c]	1.328 ± 0.170 [b]	1.440 ± 0.169 [a]	1.013 ± 0.056 [c]	**< 0.001**
**CSF Biomarkers**					
Aβ42 (Mean ± SD)	1372 ± 351[a]	759 ± 359 [b]	746 ± 263 [b]	1443 ± 403 [a]	**< 0.001**
p-Tau181 (Mean ± SD)	16.9 ± 4.6 [c]	20.0 ± 4.4 [b]	41.6 ± 13.8 [a]	33.9 ± 9.1 [a]	**< 0.001**
GAP-43 (Mean ± SD)	4028 ± 1829 [b]	3948 ± 1699 [b]	7307 ± 3153 [a]	7536 ± 2784 [a]	**< 0.001**

Numerical data are presented as the mean (standard deviation), while categorical data are expressed as the number (percentage of the total). For numerical variables, p-values were calculated using the Kruskal-Wallis test. For categorical variables, p-values were derived from contingency table analyses using the chi-square test. Post-hoc comparisons are reported as a>b>c>d.

Supplementary Table 2. Spearman's correlations among CSF and PET biomarkers.

**Variable**	Aβ42	p-Tau181	Amyloid PET SUVR	GAP-43
Aβ42	-	−0.287***	−0.689***	−0.011
p-Tau181	−0.287***	-	0.576***	0.760***
Amyloid PET SUVR	−0.689***	0.576***	-	0.288***
GAP-43	−0.011	0.760***	0.288***	-

Values are Spearman's rho correlation coefficients. Statistical significance is reported as * $P < .05$ ** $P < .01$ *** $P < .001$

Supplementary Table 3. Linear multiple regression, dependent variable: GAP43 (N = 98, GMC)

**Predictor Variables**	**β**	**t**	**F**	**Adj. R^2**
			75.721***	0.794
Age	0.088	1.820		
Education	0.061	1.307		
APOE ε4	-0.033	-0.657		
CSF Aβ42	0.349	6.879***		
CSF p-Tau181	0.894	17.650***		

Lienar regression models predicting GAP43 levels from demographic and biological variables.

* $p < .05$ ** $p < .01$ *** $p < .001$

Supplementary Table 4. Linear multiple regression, dependent variable: GAP43 (N = 787, ADNI)

**Predictor Variables**	**β**	**t**	**F**	**Adj. R^2**
			190.790***	0.547
Age	0.048	1.883		
Education	0.025	1.053		
APOE ε4	0.040	1.377		
CSF Aβ42	0.189	6.666***		
CSF p-Tau181	0.760	29.106***		

* $p < .05$ ** $p < .01$ *** $p < .001$

Supplementary Table 5. Linear multiple regression, dependent variable: GAP43 (N = 62, GMC)

**Predictor Variables**	**β**	**t**	**F**	**Adj. R^2**
			41.268***	0.767
Age	0.142	2.202*		
Education	0.017	0.255		
APOE ε4	-0.087	-1.264		
Aβ Centiloid	-0.286	-3.689***		
CSF p-Tau181	0.946	12.260***		

* $p < .05$ ** $p < .01$ *** $p < .001$

Supplementary Table 6. Linear multiple regression model, dependent variable: GAP43 (N = 777, ADNI)

**Predictor Variables**	**β**	**t**	**F**	**Adj. R^2**
			179.746***	0.535
Age	0.026	1.002		
Education	0.019	0.762		
APOE ε4	-0.009	-0.341		
Aβ PET SUVr	-0.143	-4.693***		
p-Tau181	0.800	27.391***		

* $p < .05$ ** $p < .01$ *** $p < .001$

Supplementary Table 7. Multicollinearity and goodness-of-fit diagnostics for the primary regression models predicting synaptic biomarker concentrations.

**Model**	**Outcome**	**N**	**VIF**	**Adj. R^2**	**AIC / BIC**	**Heteroscedasticity χ^2 (P)**	**Shapiro-Wilk W (P)**
Model I: CSF Aβ42 + p-tau181	GAP-43	109	1.09	0.805	135.8 / 146.6	8.43 (.015)	0.987 (.389)
Model I: CSF Aβ42 + p-tau181	Neurogranin	109	1.09	0.749	163.7 / 174.4	4.77 (.092)	0.975 (.036)
Model I: CSF Aβ42 + p-tau181	SNAP-25	109	1.09	0.814	130.9 / 141.7	8.59 (.014)	0.986 (.306)
Model II: Aβ PET Centiloid + p-tau181	GAP-43	69	1.49	0.769	99.5 / 108.4	4.63 (.099)	0.972 (.116)
Model II: Aβ PET Centiloid + p-tau181	Neurogranin	69	1.49	0.753	104.2 / 113.2	0.80 (.669)	0.960 (.026)
Model II: Aβ PET Centiloid + p-tau181	SNAP-25	69	1.49	0.782	95.7 / 104.6	4.45 (.108)	0.973 (.143)
Model I (ADNI replication): CSF Aβ42 + p-tau181	GAP-43	787	1.09	0.546	1617.6 / 1636.2	212.31 (<.001)[a]	0.890 (<.001)
Model II (ADNI replication): Aβ PET SUVR + p-tau181	GAP-43	777[b]	1.37	0.536	1613.5 / 1632.1	174.40 (<.001)[a]	0.881 (<.001)

Multicollinearity and goodness-of-fit diagnostics for the primary regression models predicting CSF synaptic biomarker concentrations. For each model we report the sample size (N), the variance inflation factor (VIF), the adjusted R^2, the Akaike and Bayesian information criteria (AIC/BIC), and tests of residual heteroscedasticity (studentized Breusch–Pagan χ^2) and normality (Shapiro–Wilk W). VIF values were well below the conventional threshold of 5 in all models (1.09–1.49), indicating no relevant multicollinearity between the amyloid and p-

tau181 predictors and supporting the interpretation of their independent associations with synaptic biomarkers. Because several models showed evidence of heteroscedasticity and mild departures from residual normality, statistical inference was based on heteroscedasticity-consistent (HC3) robust standard errors. Model I: CSF Aβ42 + CSF p-tau181; Model II: amyloid PET (Centiloid in GMC, SUVR in ADNI) + CSF p-tau181.

[a] In the ADNI cohort, the large sample size increases the sensitivity of the Breusch–Pagan and Shapiro–Wilk tests; robust HC3 standard errors were applied accordingly. [b] N differs owing to missing amyloid PET data.

Supplementary Table 8. Linear multiple regression, dependent variable: GAP43 (N = 98, GMC)

**Predictor variables**	**β**	**t**	**F**	**Adj. R^2**
			68.02*	0.779
Age	0.098	1.934		
Sex	0.031	0.630		
APOE ε4	−0.046	−0.872		
CSF Aβ42	0.254	4.879***		
CSF total tau	0.881	16.971***		

* p < .05 ** p < .01 *** p < .001

Supplementary Table 9. Linear multiple regression, dependent variable: GAP43 (N = 62, GMC)

**Predictor variables**	**β**	**t**	**F**	**Adj. R^2**
			36.53*	0.748
Age	0.178	2.632*		
Sex	0.098	1.496		
APOE ε4	−0.087	−1.228		
Amyloid PET Centiloid	−0.152	−1.975*		
CSF total tau	0.875	11.780***		

* p < .05 ** p < .01 *** p < .001

Supplementary Table 10. Sensitivity analysis including global cognitive performance (MMSE) in the adjusted linear regression model (N = 109, GMC cohort).

**Predictor variables**	**β**	**t**	**F**	**Adj. R^2**
			90.25*	0.821
Age	0.095	2.105*		
Sex	0.001	0.017		
MMSE	0.120	2.637**		
CSF Aβ42	0.298	6.455***		
CSF p-Tau181	0.946	19.898***		

* p < .05 ** p < .01 *** p < .001

Supplementary Table 11. Sensitivity analysis restricted to amyloid-positive participants (ADNI cohort).

**Model**	**Predictor**	**N**	**β**	**95% CI**	**P**	**Adj. R^2**
Model I: CSF Aβ42 + p-tau181	Aβ42	427	0.12	0.06 to 0.18	<.001	0.519
Model I: CSF Aβ42 + p-tau181	p-tau181	427	0.71	0.63 to 0.80	<.001	0.519
Model II: Aβ PET SUVR + p-tau181	PET SUVR	422	−0.08	−0.17 to 0.01	.085	0.510
Model II: Aβ PET SUVR + p-tau181	p-tau181	422	0.73	0.65 to 0.82	<.001	0.510

Supplementary Table 12. Mediation analysis including total tau as mediator of the relationship between amyloid and GAP43 (N = 69, GMC).

**Path**	**Estimate**	**95% CI**	**p**
Aβ PET Centiloid → CSF total tau (a)	0.558	0.287 to 0.829	<0.001
CSF total tau → CSF GAP43 (b)	0.844	0.683 to 1.006	<0.001
Total effect (c)	0.279	0.031 to 0.528	0.028
Direct effect (c′)	−0.192	−0.356 to −0.028	0.022
Indirect effect (a × b)	0.471	0.285 to 0.675	<0.001

Supplementary Figures

Supplementary Figure 1. Scatterplot of GAP43 versus Aβ PET Centiloid and pTau181 (GMC cohort)

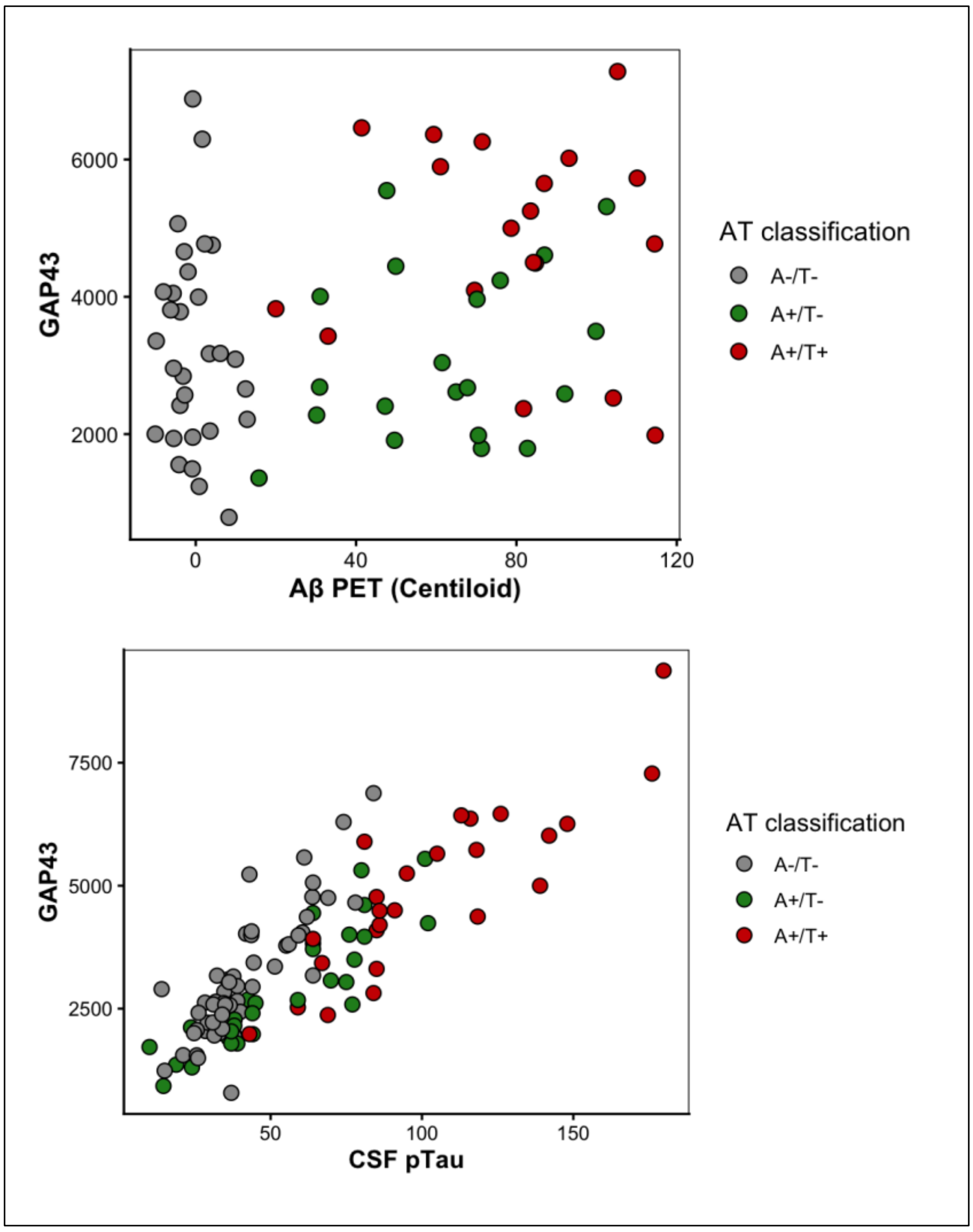

Scatterplots illustrating the relationships between CSF GAP43 and Aβ PET Centiloid (top) or CSF pTau181 (bottom), stratified according to the A/T status.

Supplementary Figure 2. Scatterplot of GAP43 versus Amyloid PET SUVR and pTau181 (ADNI cohort).

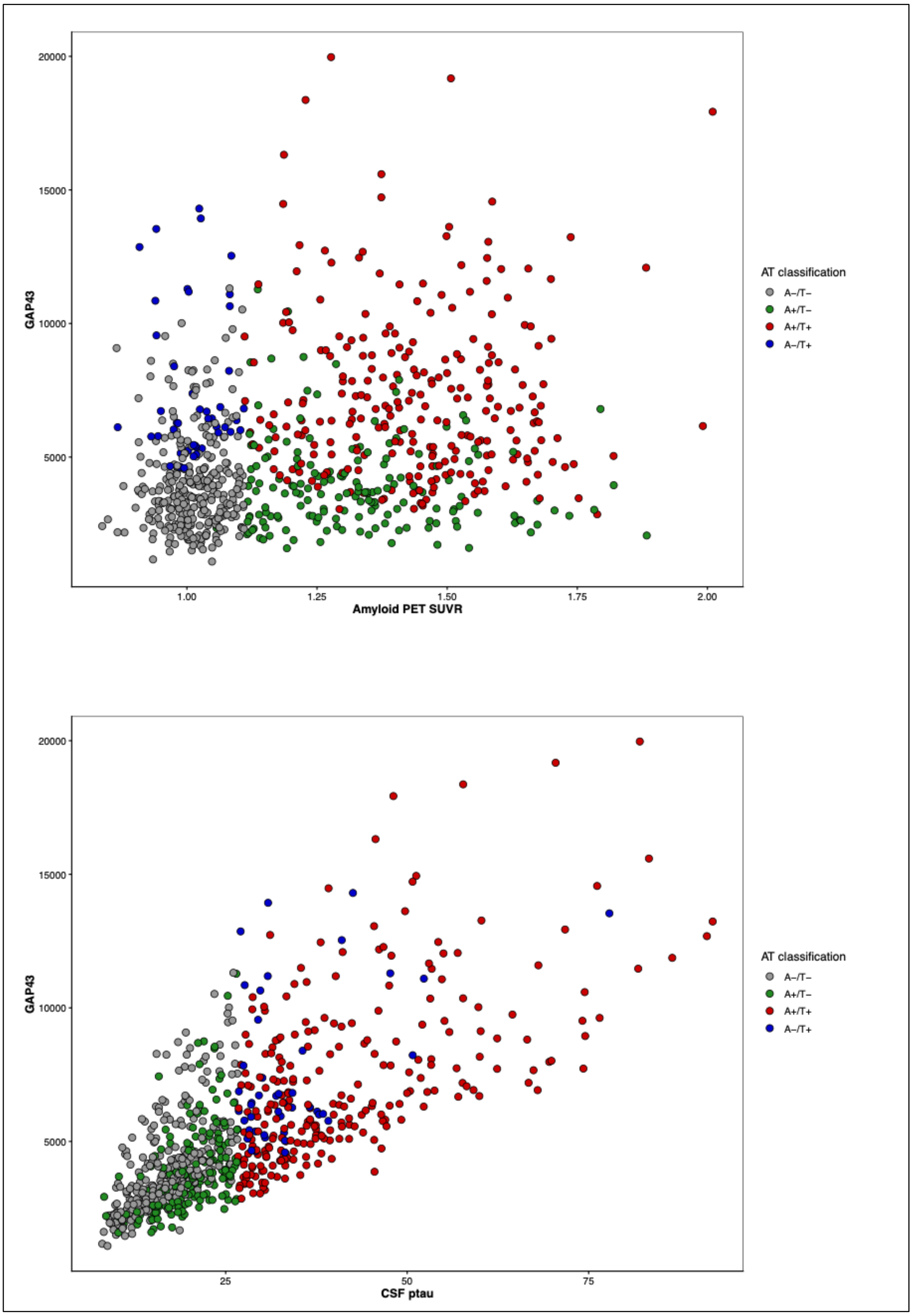

Scatterplots illustrating the relationships between CSF GAP43 and Amyloid PET SUVR Aβ42 (top) or CSF pTau181 (bottom), stratified according to the A/T status.

Supplementary Figure 3. Associations of CSF Aβ42 and tau PET global meta-ROI with GAP-43: partial residual plots (GMC cohort).

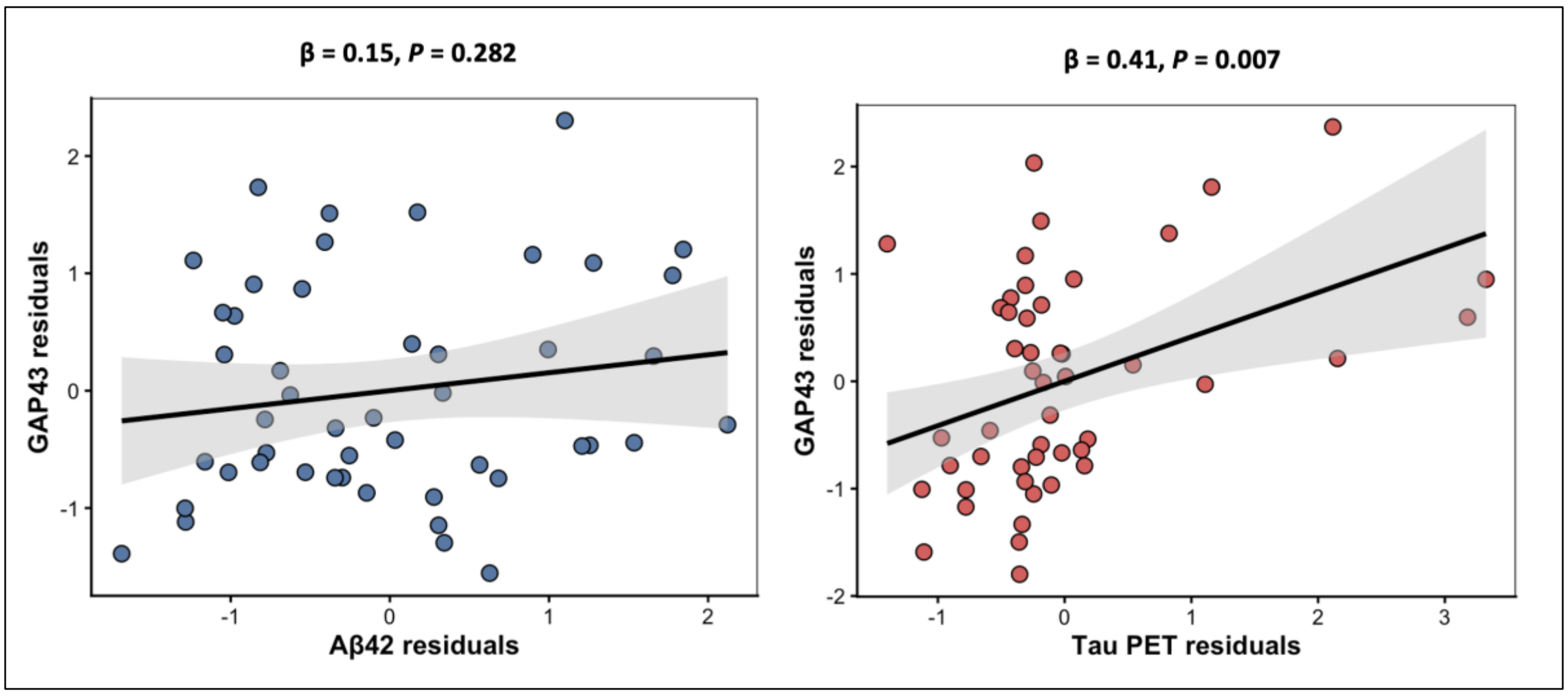

Partial residual plots illustrating the independent associations of CSF Aβ42 (left) and Tau PET global meta-ROI (right) with GAP43 after controlling for the other biomarker. Higher Tau PET global meta-ROI was associated with higher GAP43, whereas amyloid PET Centiloid did not show significant association in this subsample.

Supplementary Figure 4. Associations of amyloid PET and Tau PET global meta-ROI with GAP43: partial residual plots (GMC cohort).

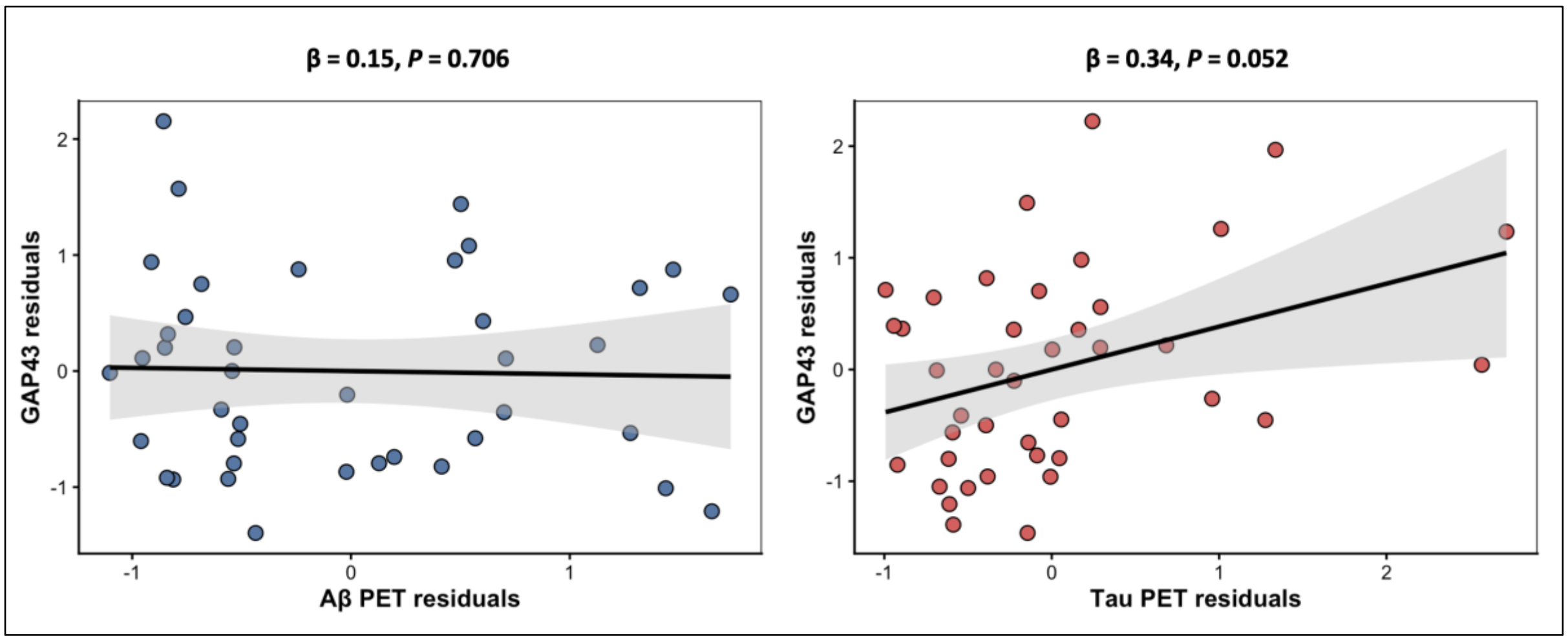

Partial residual plots illustrating the independent associations of amyloid PET Centiloid (left) and Tau PET global meta-ROI (right) with CSF GAP43 after controlling for the other biomarker. Higher Tau PET global meta-ROI was associated with higher GAP43, whereas amyloid PET Centiloid did not show significant association in this subsample.
